# Trigonal antiprismatic Co(ii) single molecule magnets with large uniaxial anisotropies: importance of Raman and tunneling mechanisms†
†Electronic supplementary information (ESI) available: Packing diagrams, detailed static and dynamic magnetic measurements, analysis of dynamic magnetic measurements and computational details. CCDC 1422285 and 1422286. For ESI and crystallographic data in CIF or other electronic format see DOI: 10.1039/c6sc02035f



**DOI:** 10.1039/c6sc02035f

**Published:** 2016-06-17

**Authors:** Yuan-Zhu Zhang, Silvia Gómez-Coca, Andrew J. Brown, Mohamed R. Saber, Xuan Zhang, Kim R. Dunbar

**Affiliations:** a Department of Chemistry , Texas A & M University , College Station , TX 77842-3012 , USA . Fax: +1-979-845-7177 ; Email: dunbar@chem.tamu.edu; b Department of Chemistry , South University of Science and Technology of China , 518055 , Shenzhen , P. R. China; c Department of Chemistry , Fayoum University , Fayoum 63514 , Egypt

## Abstract

The air-stable mononuclear Co(ii) compounds [Co^II^(Tpm)_2_][ClO_4_]_2_ (**1**, Tpm = tris(pyrazol-1-yl)methane), [Co^II^(Tpm)_2_][BPh_4_]_2_·2MeCN (**2**) with trigonal antiprismatic geometry (trigonally elongated octahedral geometry) are reported. Magnetic and theoretical studies reveal that the complexes exhibit single-molecule magnet behavior with uniaxial anisotropy and a huge energy difference between ground and first excited Karmers' doublets (∼200 cm^–1^). Under applied DC fields, compounds **1** and **2** exhibit frequency and temperature dependence of the imaginary susceptibility. The fit of the data to an Orbach relaxation process yields effective energy barriers of 30.6(1) and 44.7(6) cm^–1^ for **1** and **2**, respectively, but there is no real state at that energy. The inclusion of tunneling, direct and Raman relaxation processes leads to the conclusion that the inclusion of an Orbach process is not required to provide a good fit to the data. More interestingly, a detailed study of the dependence of the relaxation time with field shows that for these Kramers' ions, tunneling is the predominant process at low temperature and that differences in the counteranion allow for a tuning of the Raman process at higher temperatures. These findings underscore the fact that large uniaxial anisotropy can be achieved in hexacoordinate Co(ii) trigonal antiprismatic complexes which is an unexplored geometry in mononuclear single molecule magnets.

## Abstract 2

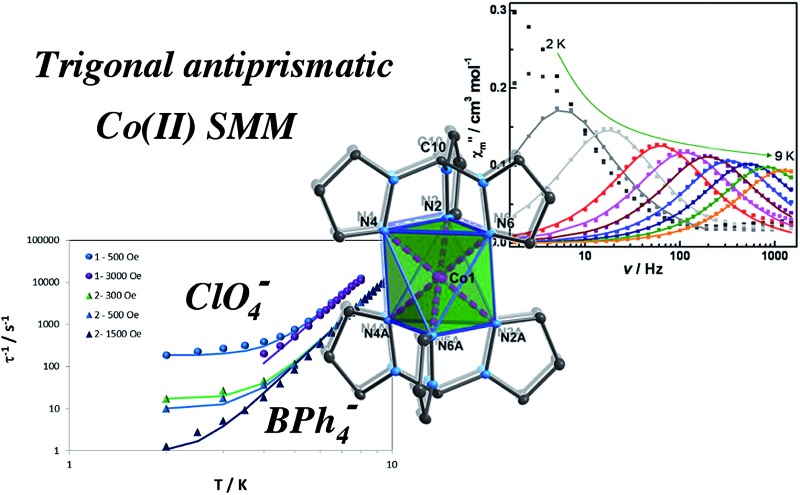
A trigonal antiprismatic Co(ii) molecule exhibits counterion-dependent relaxation of the magnetization due to the importance of the Raman relaxation process.

## Introduction

Single molecule magnets (SMMs) are molecular nanomagnets with promising applications in high density data storage, molecular spintronics, and quantum computing.^1^ Mononuclear SMMs are an emerging class of SMMs that present several advantages over higher nuclearity SMMs including the fact that a homologous series of compounds can be readily prepared that can be subjected to detailed theoretical analyses. Moreover, their anisotropies can be tuned through their coordination number, geometry and electronic structure.^2^ In considering specific target molecules, however, it is important to realize that Jahn–Teller distortions and mixing of excited states can lead to deviations in the structures and properties. These challenges notwithstanding, a great deal of effort is being directed at both experimental and theoretical analysis of transition metal mononuclear SMMs and the field is expanding rapidly.^3–6^

In 2010, Long and co-workers reported SMM behavior for a mononuclear Fe(ii) complex in a trigonal pyramidal geometry,^7^ and later reported that a linear two-coordinate Fe(i) complex exhibits the remarkably high energy barrier of –226 cm^–1^.^8^ Subsequently, a number of other mononuclear transition metal SMMs have been isolated, among which are those containing Co(ii) ions. For a series of mononuclear tetra-coordinate (tetrahedral or trigonal pyramidal) Co(ii) complexes, the anisotropy trends reveal a remarkable range of zero-field splitting parameters from *D* = –161 to +16 cm^–1^ depending on the degree of distortion and ligand field contributions.^2,9–19^ In contrast, Co(ii) SMMs with other coordination environments, *e.g.*, tri-,^20^ penta-,^21–23^ hexa-,^24–33^ hepta-^34,35^ and octa-coordinated,^36^ are less common.

Given that most Co(ii) based single chain magnets^37,38^ and SMMs^39^ are composed of octahedral Co(ii) building units, which is the most common geometry for this metal ion, the development of additional mononuclear Co(ii) SMMs requires a deeper understanding of magneto–structural correlations. Most examples in this vein exhibit large positive *D* values. For example, Pardo and co-workers first reported the highly distorted octahedral Co(ii) compound Co^II^(dmphen)_2_(NCS)_2_ with an overall rhombic (C_2V_) distortion.^24^ The compound exhibits strong positive axial magnetic anisotropy with *D* = +98 cm^–1^. Later, Trávníček and co-workers demonstrated that a Co(ii) center in a compressed octahedral geometry also gives rise to a large positive *D* value of +48(2) cm^–1^.^30^ For these cases, the barriers are assumed to be governed by the transverse anisotropy parameter, *E* (*xy* or easy-plane), which is often of a much smaller magnitude than the axial parameter, *D*_*z*_. Also Luis, Ruiz and co-workers reported a slightly elongated octahedral compound Co^II^(acac)_2_(H_2_O)_2_, (acac = acetylacetonate), with a *D* value of +57 and +63.3 cm^–1^ obtained from the fit of the magnetization curve and CASPT2-RASSI calculations respectively. In this case, the slow relaxation of the spin under an applied field was explained by direct (induced by the hyperfine interaction, *I* = 7/2) and Raman spin-phonon processes.^28^

In terms of trigonal molecules, anions such as [(Tp^R^)Fe^III^(CN)_3_]^–^ (Tp^R^ = tris/tetra(pyrazoyl)borate) and [(triphos)Re^II^(CN)_3_]^–^ exhibit significant uniaxial anisotropy^40^ and have been widely used for the elaboration of SMMs^41–50^ and SCMs.^51–53^ Recently, Gao and co-workers reported a family of Co^II^Co^III^_3_ chiral star-like compounds where the sole Co(ii) ion is located in a distorted trigonal prismatic geometry. These authors demonstrated that the magnetic anisotropy and energy barrier can be finely tuned in this family of compounds by the selection of the counter cation and the peripheral ligand substituents, achieving a huge anisotropy (*D* = –115 cm^–1^), and an impressive barrier of 109 K under a zero dc field.^29,31^ Ruiz and co-workers characterized another trigonal prismatic Co(ii) complex with *D* values of –72 and –141 cm^–1^ determined by a fit of magnetization curve and from CASSCF-RASSI calculations respectively.^2^ Another interesting case is the work of Novikov and co-workers who reported a trigonal prismatic Co(ii) cage complex with relaxation of the spin being observed at zero dc field and *D* values of –82, –109 and –110 cm^–1^ determined by a fit of magnetic susceptibility, NMR spectroscopy and from CASSCF/NEVPT2 calculations respectively.^32^ An ongoing interest in this coordination geometry is apparent from recent literature.^32,54^

**Fig. 1:**
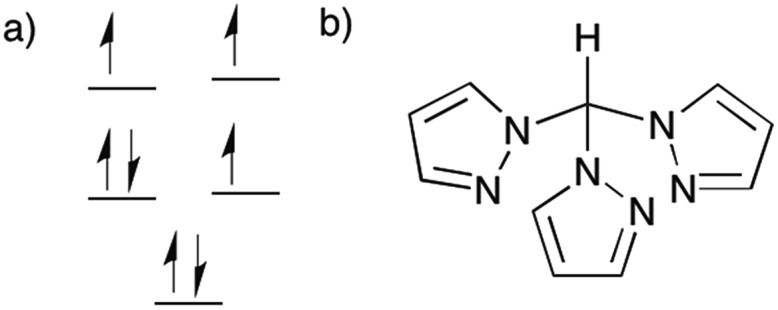
(a) Scheme of the orbital splitting for **1** and **2**. (b) Tpm ligand.

In light of these aforementioned results, we have focused on Co(ii) compounds with trigonal antiprismatic geometry that are less sterically rigid but which exhibit a similar splitting of the d-orbitals (Fig. 1a) as the trigonal prismatic geometry which was previously calculated at the DFT level.^55^ The presence of three electrons in two nearly degenerate e_g_ orbitals is expected to lead to a large anisotropy, in this case it produces a uniaxial magnetic anisotropy as confirmed by EPR analysis 50 years ago.^56^ Thus far, however, no SMMs have been reported with this geometry. We postulated that *fac*-tripodal ligands could enforce a trigonal antiprismatic geometry, thus we undertook a systematic study of Co(ii) complexes equipped with tris(pyrazol-1-yl)methane (Fig. 1b), a ligand that has been used in spin crossover compounds.^57–65^ Herein, we report two mononuclear SMMs, *viz.*, [Co^II^(Tpm)_2_][ClO_4_]_2_ (**1**) and [Co^II^(Tpm)_2_][BPh_4_]_2_·2MeCN (**2**), which have the same magnetic unit, [Co^II^(Tpm)_2_]^2+^, but different chemical surroundings. Magnetic studies indicate that both compounds possess a large energy difference between the ground and excited states (large energy barrier) and field-induced relaxation of the magnetization.

## Experimental

### Synthetic procedures

#### Starting materials

The Tpm ligand was synthesized according to a published procedure.^66^ [Co(H_2_O)_6_][ClO_4_]_2_ (Acros), [Co(H_2_O)_6_]Cl_2_ (Acros), NaBPh_4_ (Sigma-Aldrich) and the solvents (Acros, reagent grade) were used as received.

#### Synthesis of [Co(Tpm)_2_][ClO_4_]_2_ (**1**)

[Co(H_2_O)_6_][ClO_4_]_2_ (73.5 mg, 0.20 mmol) in methanol (6 mL) was added to an acetonitrile (6 mL) solution of Tpm (98.5 mg, 0.46 mmol). The resulting yellow solution was stored in an open atmosphere for 3 days. Yellow platelet crystals of **1** were collected *via* filtration and dried in air for 5 min. Yield: 98.2 mg, 71%. Anal. calcd C_20_H_20_Cl_2_CoN_12_O_8_ (F.W. = 686.31 g mol^–1^): C, 35.00; H, 2.94; N, 24.49. Found: C, 35.23; H, 2.98; N, 24.57.

#### Synthesis of [Co(Tpm)_2_][BPh_4_]_2_·2MeCN (**2**)

Treatment of [Co(H_2_O)_6_]Cl_2_ (47.5 mg, 0.20 mmol) with NaBPh_4_ (137 mg, 0.40 mmol) in acetonitrile (6 mL) for 30 minutes produced a white precipitate of NaCl which was removed by filtration. The filtrate was added to 6 mL of a methanol solution of Tpm (96.3 mg, 0.45 mmol). The resulting yellow solution was stored in air for 3 days during which time yellow platelet crystals had formed. Yield: 158 mg, 65%. Anal. calcd C_72_H_66_B_2_CoN_14_ (F.W. = 1207.94 g mol^–1^): C, 71.59; H, 5.51; N, 16.23. Found: C, 71.59; H, 5.70; N, 16.33.

### Materials and methods

#### Physical measurements

Single crystal X-ray data for **1** and **2** were collected on a Bruker APEX-II diffractometer equipped with a CCD detector at 110 K. Direct current (dc) and alternating current (ac) susceptibility measurements for samples of crushed crystals immobilized in eicosane and sealed in a quartz NMR tube were performed on a Quantum Design SQUID, model MPMS XL-7. Diamagnetic corrections were applied using Pascal constants. Elemental analyses were performed by Atlantic Microlab, Inc.

#### Computational details

Single point calculations of compounds **1** and **2** were performed using the crystallographic geometries provided in the cif files. The *ab initio* calculations were performed using the two-step approach implemented in the ORCA 3.0.3 program in which the spin–orbit coupling (SOC) and spin–spin coupling (SSC) relativistic effects are included *a posteriori*.^67^ The electronic configuration of Co(ii) is d^7^, so the selected active space CAS(7,5) contains 7 electrons in the 5 essentially atomic d orbitals. To evaluate the effects of the dynamic correlations, N-Electron Valence Perturbation Theory (NEVPT2) was employed. The Karlsruhe polarized triple-ζ basis set (TZVP)^68^ and the auxiliary def2-TZV/J basis set^69^ for resolution of identity (RI) approximation were employed.

### Crystallography

**Fig. 2:**
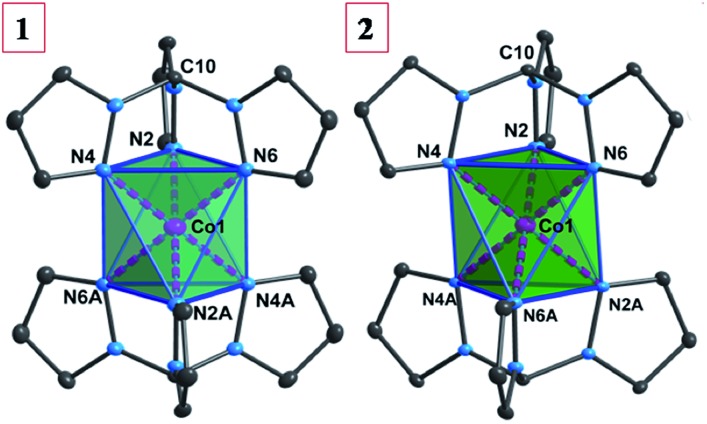
The molecular structures of **1** and **2** (thermal ellipsodes are at 30% level and all counter anions and hydrogen atoms have been omitted for clarity).

Compounds **1** and **2** crystallize in the monoclinic *P*2_1_/*n* space group with the Co(ii) ion residing on an inversion center (Fig. 2). Selected crystallographic data are available in Table 1. The Co(ii) ion is coordinated to two tripodal ligands of Tpm in a trigonally distorted octahedral geometry namely that of an elongated trigonal antiprism. Selected bond distances and angles are listed in Table 2. The Co–N bond distances are nearly equal and are in the range of 2.107(3)–2.112(3), 2.101(1)–2.110(1) Å for **1** and **2**, respectively. The intra-ligand bite angles (N_Tpm_–Co–N_Tpm_) are acute and range from 83.68(13)–84.71(13)° for **1**, and 83.99(4)–85.53(4)° for **2**, respectively. The inter-ligand *cis* N_Tpm_–Co–N_Tpm′_ angles are obtuse: 95.09(13)–96.32(13)°, and 94.47(4)–96.10(4)° for **1** and **2**, respectively. The trigonal elongation is further evidenced by the N–N distances within the base faces (faces N2–N4–N6 and N2A–N4A–N6A, 2.815(9)–2.848(2) Å for **1**; 2.814(1)–2.861(1) Å for **2**), which are significantly shorter than the N–N distances (N2–N2A/N4A, N4–N4A/N6A, N6–N6A/N2A; 3.110(10)–3.144(4) Å for **1**; 3.094(1)–3.125(1) Å for **2**). As a result, the distances (*d*_b_) between the two bases are 2.658(3) and 2.641(2) Å, respectively, significantly longer than those (*d*_s_) for the three pairs of side faces, 2.335(3)–2.370(2) Å for **1**, 2.327(3)–2.371(4) Å for **2**, giving *δ* = *d*_b_ – *d*_s_ as 0.311, 0.293 Å (*δ* = 0 for an ideal octahedral geometry). In addition, a geometrical analysis was performed with the help of the SHAPE program.^70,71^ The continuous shape measurements (*S*) for compounds **1** and **2** give, respectively, values of 36.133, 35.861 for ideal octahedral geometry and 24.843, 24.680 for ideal trigonal prismatic geometry. These measurements confirm that the geometry of both compounds is far from the ideal octahedral or trigonal prism geometries (*S* = 0 for the perfect geometry). This is not uncommon for complexes with two claw-type tridentate ligands, that form an elongated trigonal antiprismatic geometry rather than an elongation distortion with a Bailar twist.^72^

**Table 1:** Selected crystallographic data for **1** and **2** at 110(2) K

Compound	**1**	**2**
Crystal system	Monoclinic	Monoclinic
Space group	*P*2_1_/*n*	*P*2_1_/*n*
*a*, Å	10.500(8)	9.7482(8)
*b*, Å	7.818(6)	17.1636(14)
*c*, Å	16.997(13)	18.9136(16)
*β*, deg	103.529()	98.5041()
*V*, Å^3^	1356.5(18)	3129.7(4)
*D*_c_, g cm^–3^	1.680	1.282
*Z*	2	2
*μ*, mm^–1^	0.900	0.330
*F*(000)	698	1266
GooF	1.078	1.039
*R*_1_ ^*a*^	0.0602	0.0323
w*R*_2_ ^*a*^	0.1635	0.0762

^*a*^ *I* ≥ 2σ(*I*): R_1_ = ∑ ∥F_o_| – |F_c_∥ / ∑ |Fo|, *w*R_2_ = {∑ [*w*(F_o_^2^ – F_c_^2^)^2^] / ∑[*w*(F_o_^2^)^2^]}^1/2^.

**Table 2:** Selected bond distances (Å) and angles (°) for **1** and **2**

Compound	**1**	**2**
Co1–N2	2.112(3)	2.110(1)
Co1–N4	2.107(3)	2.104(1)
Co1–N6	2.107(3)	2.101(1)
N2–N4	2.815(9)	2.861(1)
N2–N6	2.845(5)	2.842(1)
N4–N6	2.848(2)	2.814(1)
N2–N4A	3.144(4)	3.094(1)
N2–N6A	3.114(3)	3.107(1)
N4–N6A	3.110(10)	3.125(1)
N2–Co1–N4	83.68(13)	85.53(4)
N2–Co1–N6	84.89(14)	84.90(4)
N4–Co1–N6	84.91(13)	83.99(4)
Bite angle avg.	84.49(13)	84.81(4)
N2–Co1–N2A	180.0	180.0
N2–Co1–N4A	96.32(13)	94.47(4)
N2–Co1–N6A	95.11(14)	95.10(4)
N4–Co1–N6A	95.09(13)	96.10(4)
Co1···C	3.117(8)	3.113(1)
Co···Co	7.818(6)	9.748(1)

The nearest Co···Co distance in **2** (9.748(1) Å) is significantly longer than that in **1** (7.818(6) Å), due to the larger [BPh_4_]^–^ anions (Fig. S1 and S2, ESI†). Two different orientations of the molecules in the crystal packing (the pseudo-*C*_3_ axes along the Co1–C10 vectors) are present with an angle of 40.8° for **1** and 69.8° for **2**, respectively.

## Results and discussion

### Static magnetic properties

**Fig. 3:**
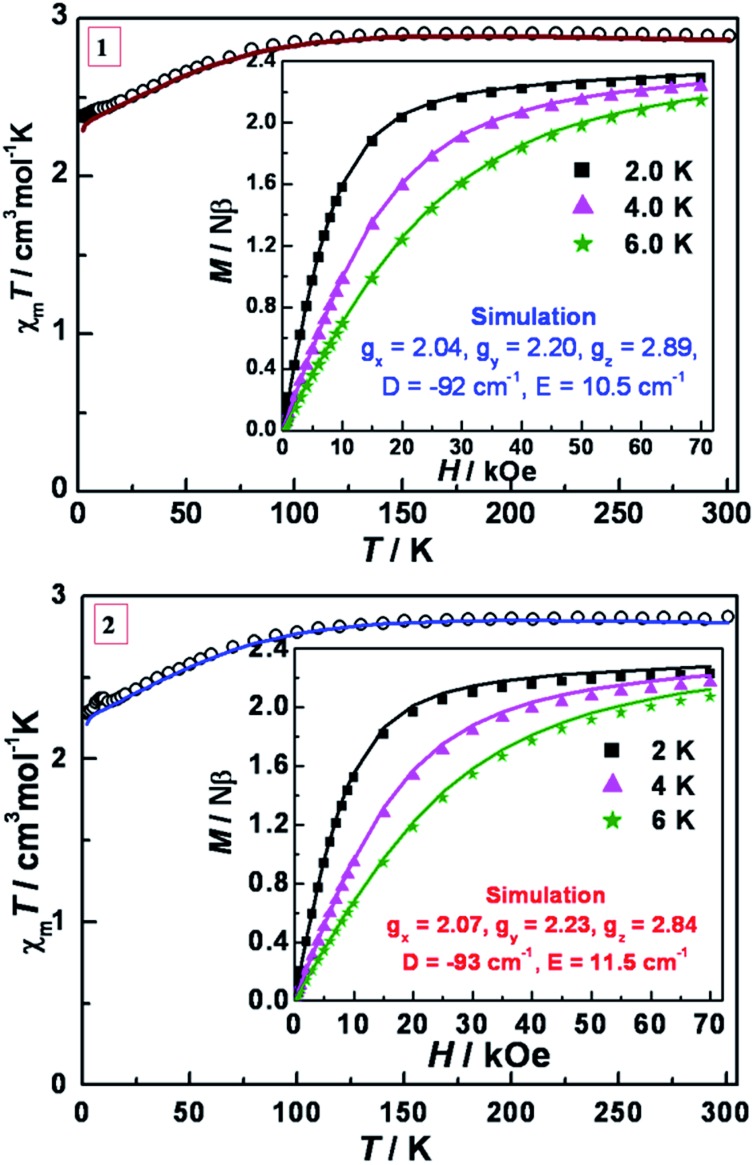
Variable-temperature dc magnetic susceptibility data in an applied field of 1 kOe for 1 (top) and 2 (bottom). Insets: *M vs. H* plots at 2, 4, 6 K. Solid lines are the best simulations obtained by the program PHI based on eqn (1).

The variable temperature magnetic susceptibility data for **1** and **2** were measured under an applied dc field of 1 kOe (Fig. 3). At 300 K, the *χ*_m_ *T* values are 2.73 and 2.81 cm^3^ mol^–1^ K for **1** and **2**, respectively, which are much higher than the spin-only value (1.875 cm^3^ mol^–1^ K) for an isotropic Co(ii) (*S* = 3/2, *g* = 2.0) ion, indicating strong spin–orbit coupling effects. As the temperature is lowered, the *χ*_m_ *T* value remains roughly constant until ∼120 K and then gradually decreases until ultimately reaching 2.38 and 2.28 cm^3^ mol^–1^ K at 2.0 K, respectively. The isothermal dc field (*H*) dependence of the magnetization (*M*) was measured up to 7 T at temperatures of 2, 4 and 6 K, respectively (inset of Fig. 3). The magnetization at 7 T (2.23–2.27 Nβ) is significantly lower than the expected saturation for a system with (*S* = 3/2 and *g* > 2) and the non-superposition of the *M vs. H*/*T* plots at higher fields (Fig. S3, ESI†) indicate the presence of considerable magnetic anisotropy.

To analyze the experimental data, the PHI program was employed using the Hamiltonian in eqn (1).^73^ This Hamiltonian is divided into three terms, the first two being related to the crystal field Hamiltonian (using the Stevens notation)^74^ with the third one representing the Zeeman Hamiltonian with an anisotropic g tensor.1
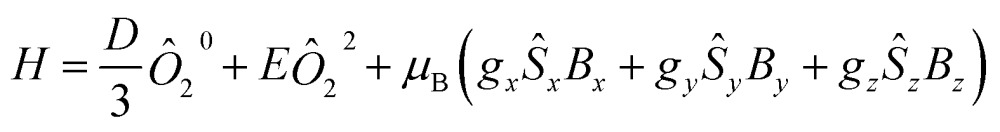
where *μ*_B_ is the Bohr magneton, *D*, *E*, *Ô*_*k*_^*q*^, *g*_*i*_, *ŝ*_*i*_, *B*_*i*_ represents the axial and rhombic zero-field splitting, the Stevens operator equivalents, the g tensor, the spin operator and the magnetic field, respectively. The best simulations for both *χ*_m_ *T vs. T* and *M*–*H* data are found to be *g*_*x*_ = 2.04, *g*_*y*_ = 2.20, *g*_*z*_ = 2.89, *D* = –92 cm^–1^, and *E* = 10.5 cm^–1^ for **1**; *g*_*x*_ = 2.07, *g*_*y*_ = 2.23, *g*_*z*_ = 2.83, *D* = –93 cm^–1^, and *E* = 11.5 cm^–1^ for **2**, respectively (Fig. 3). It should be mentioned that using a positive *D* value cannot reproduce the experimental data but it is difficult to obtain accurate values of *D* and *E*, as has been noted for other compounds with huge negative *D* values, which are too large to be determined by high-field EPR spectroscopy. The *D* values are among the largest negative ones reported for mononuclear transition metal complexes^3–5^ and comparable to those for the Co(ii) ion in a trigonal prismatic geometry.^29,31,32^

To further verify the obtained values, *ab initio* calculations using the experimental geometry and the CASSCF/NEVPT2 approach with the Orca code were performed.^67^ The calculated energy of the first spin-free states is shown in Table S15.† The energy difference between the ground and first excited states is very small (73.9 and 53.1 cm^–1^, for **1** and **2** respectively, at NEVPT2 level) while the second excited states is almost 3000 cm^–1^ above the ground state. These findings are in accord with the first two states being nearly degenerate indicating that the Jahn–Teller effect is small and the first order SOC should be important.

The SOC mixing of the ground and excited states gives rise to four Kramers' doublets (KD) relatively close in energy (Table 3). For a Fe(ii) system with ^5^E ground state in which the distortions can change the energy and lift the orbital degeneracy, a more complicated Spin Hamiltonian (SH) with a larger number of parameters has been proposed.^75^ This point notwithstanding, the effective SH employed in the experimental part gives us a qualitative estimation of the large energy difference between states (|2D| equal to 184 and 186 cm^–1^ for **1** and **2** respectively), which is corroborated by the theoretical calculations (232.8 cm^–1^ for **1** and **2**). Although this result does not provide information about the proximity in energy of the third and fourth KD, from the theoretical calculations one can conclude that the population of these two KD at 300 K is smaller than 5% (Table S11†) and that the effective Hamiltonian employed in the experimental part provides a reasonable estimation. Using the effective Hamiltonian implemented in Orca, the *D* and *E* values match very well with the experimental ones (Table S12†). The analysis of the principal contribution to the *D* value revealed that the origin is from the first excited state while the major contribution to *E* emanates from the second excited state (Table S13†).

**Table 3:** Relative energy (*E*) in cm^–1^ of the six lowest Kramers' doublets (KD) computed at the NEVPT2 level and the corresponding effective *g*_*i*_ values for each doublet projected on an *S* = 1/2 pseudo spin for **1** and **2**

KD	**1**				**2**			
	*E*	*g*_*x*_	*g*_*y*_	*g*_*z*_	*E*	*g*_*x*_	*g*_*y*_	*g*_*z*_
1	0	0.71	0.73	8.93	0.0	0.72	0.74	8.92
2	232.8	0.68	1.50	4.78	236.3	0.38	1.21	4.81
3	509.1	0.86	1.02	1.21	504.2	0.74	0.85	0.95
4	809.8	0.06	0.08	3.05	817.2	0.06	0.08	3.06
5	3290	0.13	0.14	6.38	2592	0.07	0.07	6.37
6	3377	2.12	3.58	3.85	2675	2.12	3.64	3.77

In addition, the effective *g*_*i*_ values (Table 3) confirm the axial character of the ground state and agree nicely with the *g*_*i*_ values reported 50 years ago for the analogous compound [Co(Tpm)_2_](NO_3_)_2_.^56^

### Dynamic magnetic properties

To probe the dynamics of the magnetization, ac magnetic susceptibility data for **1** and **2** were collected as a function of both temperature and frequency in the presence and absence of an applied external dc field. Under a zero dc field, no out-of-phase ac signal was observed. This behaviour is not uncommon in mononuclear SMMs and has been attributed to tunnelling, dipolar interactions and/or hyperfine interactions. In the case of Kramers' ions, direct transitions are forbidden under pure electric fields (van Vleck cancellation),^76,77^ but electric fields such as hyperfine or dipolar interactions can make the transitions allowed.^78^ Under applied dc fields both **1** and **2** display typical slow relaxation of the magnetization as has been observed for other molecules (Fig. S4 and S13†).

For compound **1**, variable-frequency ac data were measured at 1.8 K under applied dc fields in the range of 0 to 5000 Oe. No *χ*′′ signal was observed at zero dc field, while a peak appeared at ∼22 Hz when a 250 Oe was applied. When a larger dc field is applied, this peak increases until 750 Oe and then decreases at higher fields. At 750 Oe a new signal appears at lower frequencies, an indication of several relaxation processes being operative at lower temperatures (Fig. S4†). Due to the superposition of the peaks and the number of points for one of the relaxation processes, not all of the data could be fit. Also, for fields larger than 1500 Oe, the *α* value is ∼0.3 indicating a large distribution of relaxation times (Fig. S5 and Table S1†), which may be related to the closer distance between molecules for **1** (7.818 Å). These values were discarded for the subsequent analysis.

Under a 500 Oe dc field, variable-temperature (2.0–15.0 K) ac susceptibilities exhibit highly frequency-dependent peaks in both the in-phase and out-of phase signals (Fig. S6†). At 100 Hz, there is an indication of two peaks, the second of which has a maximum at ∼5 K. To gain more insight into the field dependence, variable-frequency ac data were measured under applied dc fields in the range of 0 to 10 000 Oe at 5.0 K (Fig. S7†). From 125 to 625 Oe, the maximum in *χ*′′ is nearly constant with a slight shift to higher frequencies. From 625 to 3000 Oe, when the field is increasing, the maximum in *χ*′′ moves to lower frequencies. For larger fields, the maximum in *χ*′′ moves to higher frequencies. The relaxation times obtained from the fittings of the Cole–Cole plots with a modified Debye model were plotted as a function of the dc fields (Fig. S8, S9 and Table S2†). The value of *τ* ^–1^ first increases up to 625 Oe, then decreases from 625 to 3000 Oe and finally increases for fields larger than 3000 Oe. The decrease up to 3000 Oe is ascribed to a tunneling process and the increase at higher fields is attributed to a direct process due to its dependence with field.

The variable-frequency ac data at different temperatures were collected under 500 and 3000 Oe (Fig. S8† and 4, respectively). At 500 Oe the maximum in *χ*′′ moves to higher frequencies with increasing temperature. The increase is very slight until 4.5 K and then becomes more pronounced at higher temperatures. Two processes are observed at 3000 Oe up to 3.5 K, but at higher temperatures only one highly frequency dependent peak is observed. The Cole–Cole plots were fitted to a modified Debye model with *α* values less than 0.1 at 500 Oe and at 3000 Oe above 4.0 K, indicative of a relatively narrow distribution of relaxation pathways (Fig. S11, S12, Tables S3 and S4†).

**Fig. 4:**
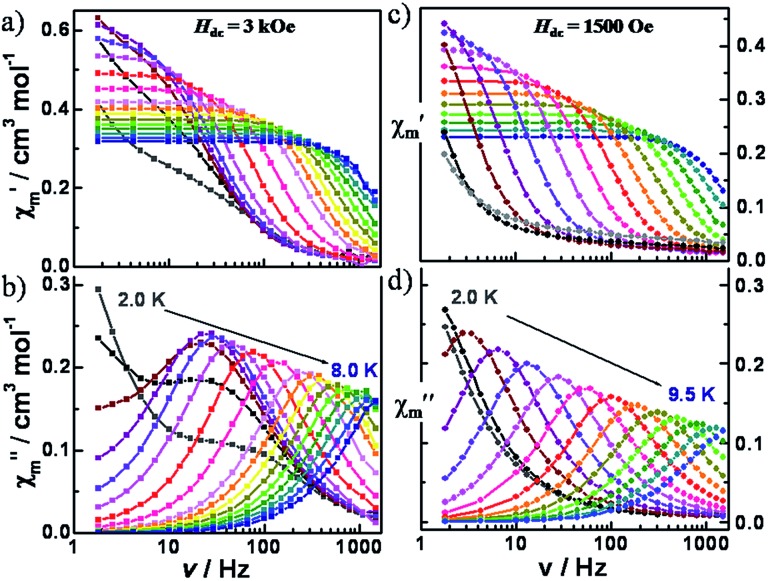
Variable-frequency in-phase (*χ*_m_′) and out-of-phase (*χ*_m_′′) components of the ac magnetic susceptibility data for **1** (left, a and b) and **2** (right, c and d), collected in a 5 Oe ac field and a dc field of 3000 Oe (**1**) and 1500 Oe (**2**), respectively, oscillating at frequencies of 1 to 1500 Hz.

For compound **2**, similar dynamic susceptibility behavior was observed. Variable-frequency ac data were measured at 1.8 K under applied dc fields in the range of 0 to 1000 Oe (Fig. S13†). Only a low frequency signal without an obvious maximum was observed. Under a 500 Oe dc field, variable-temperature (2.0–15.0 K) ac susceptibility data showed highly frequency-dependent peaks in both the in-phase and out-of phase signals (Fig. S14†). At 100 Hz, a maximum is observed at 6 K. To gain more insight into the field dependence, the variable-frequency ac data were measured under applied dc fields in the range of 0 to 10 000 Oe at 6.0 K (Fig. S15†). The relaxation times obtained from the fittings of the Cole–Cole plots with a modified Debye model were plotted as a function of the dc fields (Fig. S16, S17 and Table S5†). It was found that the relaxation times increase up to a dc field of 1500 Oe and then decrease at higher dc fields. The first decrease of *τ* ^–1^ can be attributed to tunneling whereas the subsequent increase is in accord with a direct process.

The variable-frequency ac data at different temperatures were collected under fields of 300, 500 and 1500 Oe (Fig. S18, S20† and 3, respectively). In this case, at the optimum field of 1500 Oe, only one highly frequency dependent peak is observed over the range of temperatures measured. The Cole–Cole plots were fit to a modified Debye model which gave small *α* values of less than 0.1 at above 4.0 K, indicative of a relatively narrow distribution of relaxation pathways (Fig. S19, S21, S22 and Tables S6–S8†).

For both complexes, the dependence of *τ* ^–1^ with the temperature at different fields was analyzed. A fit with the Arrhenius law (*τ* = *τ*_0_ exp(*U*_eff_/*k*_B_ *T*)), corresponding to the thermally activated relaxation *via* an Orbach process (Fig. S23†) was performed leading to effective energy barriers (*U*_eff_) and pre-exponential factors (*τ*_0_) of 30.6(7)–33.6(5) cm^–1^/2.0(2)–3.3(7) × 10^–7^ s for **1**; 42.5(6)–44.7(6) cm^–1^/1.0(1)–1.5(2) × 10^–7^ s for **2**, respectively. These values, however, are far from the energy difference between ground and excited states (∼200 cm^–1^). This discrepancy and the absence of a real state necessary for an Orbach process at 30–40 cm^–1^ are clear indications of the presence of other relaxation processes.

Previously the spin relaxation of other mononuclear SMMs has been fitted considering direct, Raman, tunneling and/or Orbach processes. For linear Fe(ii) complexes, the dependence of *τ* with field was fitted to obtain direct and tunneling parameters. The dependence of *τ* with temperature was fitted by considering direct, Raman, tunneling and Orbach processes but by fixing the previously obtained parameters.^79^ A similar procedure was employed for a trigonal prismatic Co(ii) cage complex.^32^ For a previously reported octahedral Co(ii) mononuclear SMM with a positive *D* value, the spin relaxation was explained by including the hyperfine coupling and the nuclear spin-lattice interaction, which allows for the one phonon (or direct) relaxation process.^28^ In that case, the variable temperature dynamic susceptibility of a magnetically diluted sample was fitted with a combination of direct and Raman (two phonon) processes. In a similar way, direct and Raman processes have been considered in order to fit the spin relaxation of other 3d compounds.^80,81^ For a recently reported four-coordinate Co(ii) compound the spin relaxation at zero dc field was fitted considering Raman and Orbach; in this case they fixed the thermal energy barrier to the value obtained from the far infrared transmission spectra to be 230 cm^–1^ and then fit the rest with the Raman contribution.^82^ In other cases the data have been fit only with a Raman term when the energy barrier did not match the energy difference between states.^26,34,83–86^ In another system, the dependence of *τ* was fit with Orbach, Raman and direct terms although in one of these cases the Raman term was found to be zero.^87^ while in the other the direct was zero.^35^

In the current study, the relaxation times were re-evaluated considering direct, Raman, tunneling and Orbach processes. For both compounds, all attempts to fit the dependence of *τ* ^–1^ with field according to eqn (S1)† (which considers direct, tunneling and a constant to include processes that are independent with field) were not successful. Clearly there is a more complex dependence of *τ* ^–1^ with field. A fit of the dependence of *τ* ^–1^ with field including all the possible relaxation pathways (eqn (S2), Fig. S24 and Table S9†) would result in an over-parameterization of the curves, especially without fixing the direct and tunneling parameters. We, therefore, adopted another approach.

The more common relaxation processes for mononuclear compounds are direct, tunneling (usually dominant at low temperatures), Raman and Orbach (usually predominant at higher temperatures).^88^ First we analyzed the high temperature region. The large energy difference between states should lead to a very slow relaxation time (slower than the measurable relaxation times with the ac measurements that are possible with our SQUID). If the energy difference between the states is larger than the Debye temperature, the Raman term will be the principal one at high temperature with a negligible Orbach process.^88^ In fact, an attempt to fit the high temperature regime with both processes with an energy barrier of 200 cm^–1^ as a starting parameter, led to the discovery that the value does not vary and the data can be fit exclusively with the Raman term. For other mononuclear SMMs, it has been observed that both processes coexist even for energy barriers larger than 200 cm^–1^. In our case, however, if we examine the contributions of Orbach and Raman terms we see that the Raman is the one which describes the shape of the curve (Fig. S25†).

Secondly, we analyzed the lower temperature region. For both compounds, *τ* ^–1^ is larger at lower fields; the values at 500 Oe *vs.* 3000 Oe for **1**, and 300, 500 and 1500 Oe for **2** were compared. These results indicate that the predominant process is tunneling because the opposite trend is expected for a direct process (*τ* ^–1^ is proportional to H^4^ for Kramers' ions).^88^ Taking this aspect into consideration and to avoid the over-parameterization of the curves, we modelled the dependence of *τ* ^–1^ with temperature using eqn (2) and S3,† which considers just tunneling and Raman terms.

2 *τ*^–1^ = *B* + *CT*^*n*^

**Fig. 5:**
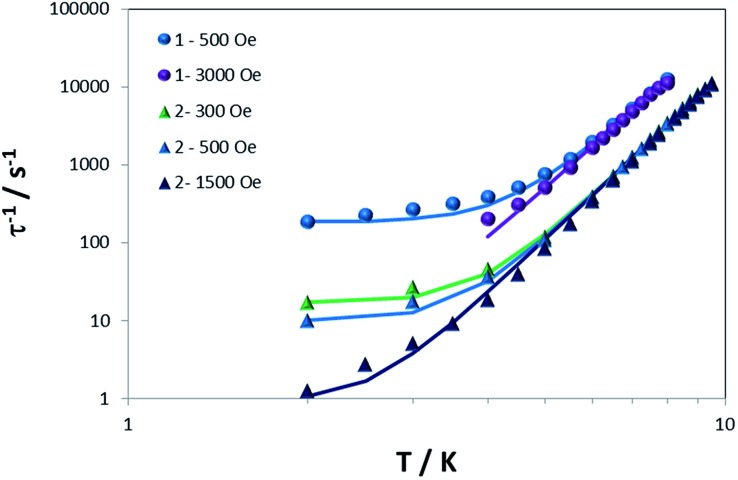
Dependence of *τ* ^–1^ with *T* for **1** and **2** under different dc fields. Solid lines are the best simulation of the curves using eqn (2).

Fig. 5 shows the best fit using eqn (2) with constraining the *B* parameter to the minimum value of *τ* ^–1^. The obtained parameters are *B* = 184 and 0 s^–1^ at 500 and 3000 Oe respectively, *C* = 0.0117 s^–1^ K^–*n*^ and *n* = 6.65 for **1** and *B* = 17.3, 9.9 and 0.9 s^–1^ at 300, 500 and 1500 Oe respectively, *C* = 0.0011 s^–1^ K^–*n*^ and *n* is 7.18 for **2**. From these parameters, one can deduce that, for an optimal field, the tunneling is essentially negligible and that the exponent for the Raman term in both cases is very close to 7. For Kramers ions, this exponent should be 9 (or 5 in the presence of low-lying states),^74^ but different values have been reported and justified by the presence of both acoustic and optical phonons.^26,30,32,82^ The Raman coefficient for **1** is one order of magnitude larger than for **2**, a reflection of how a change in counterion modifies the spin-lattice Raman process.

As revealed by the experimental magnetic data and the theoretical calculations, the anisotropy for both compounds is very similar so the differences in the dynamic susceptibility behavior between **1** and **2** are attributed to the different counter anion. When we move from a smaller anion in **1** (ClO_4_^–^) to a larger one in **2** (BPh_4_^–^) there is an (i) increase in the distance between Co(ii) centers which reduces the dipolar interactions, and (ii) a change in the crystal packing and chemical environment of the SMM unit.

In the case of **1** there is, in general, a larger the distribution of relaxation times (*α*) and two different processes were observed at lower temperatures and different dc fields, most likely due to the dipolar interactions because of the closest contact being 7.818 Å. In both of the current complexes we have seen from the dependence with field that tunneling is the predominant process at low temperature (whereas for other Co(ii) mononuclear SMMs the low temperature behavior is attributed to direct). In addition, the change in the crystal packing and chemical environment due to the larger anion in **2** leads to a decrease in the Raman parameter (*C*). A deeper understanding of this process will allow us to tune this effect with the appropriate election of the chemical environment. It is not unreasonable to expect that further modifications of this geometry may result in a compound with an extremely high barrier as observed for other geometries.

## Conclusions

This study reports the first examples of six-coordinate trigonal antiprismatic Co(ii) complexes with SMM behavior. Of particular note is the fact that the SMMs [Co(Tpm)_2_][ClO_4_]_2_ and [Co(Tpm)_2_][BPh_4_]_2_·2MeCN exhibit some of the largest uniaxial anisotropies reported for transition metal complexes to date. The effective energy barriers, however, are smaller than 45 cm^–1^ and there is no real state at that energy. Our detailed study of the dynamic susceptibility measurements revealed that other relaxation processes are taking place, with tunneling and Raman being the predominant ones at low and high temperatures respectively. In addition, there is a large dependence of the Raman process on the identity of the counterion. The findings underscore the point that, in order to design mononuclear SMMs with improved properties, it is imperative to elucidate the different relaxation processes and to be able to understand why a particular one dominates over the others. It is therefore crucial to obtain detailed dynamic susceptibility measurements for characterizing the magnetic behaviour of such systems. Ongoing efforts are being directed at designing new members of this family with different tripodal ligands to improve the magnetic properties and realize a system with zero-field slow relaxation.

## References

[cit1] Gatteschi D., Sessoli R. (2003). Angew. Chem., Int. Ed. Engl..

[cit2] Gómez-Coca S., Cremades E., Aliaga-Alcalde N., Ruiz E. (2013). J. Am. Chem. Soc..

[cit3] Gómez-Coca S., Aravena D., Morales R., Ruiz E. (2015). Coord. Chem. Rev..

[cit4] Craig G. A., Murrie M. (2015). Chem. Soc. Rev..

[cit5] Bar A. K., Pichon C., Sutter J. P. (2016). Coord. Chem. Rev..

[cit6] Atanasov M., Aravena D., Suturina E., Bill E., Maganas D., Neese F. (2015). Coord. Chem. Rev..

[cit7] Freedman D. E., Harman W. H., Harris T. D., Long G. J., Chang C. J., Long J. R. (2010). J. Am. Chem. Soc..

[cit8] Zadrozny J. M., Xiao D. J., Atanasov M., Long G. J., Grandjean F., Neese F., Long J. R. (2013). Nat. Chem..

[cit9] Buchholz A., Eseola A. O., Plass W. (2012). C. R. Chim..

[cit10] Boča R., Miklovič J., Titiš J. (2014). Inorg. Chem..

[cit11] Huang W., Liu T., Wu D., Cheng J., Ouyang Z. W., Duan C. (2013). Dalton Trans..

[cit12] Idešicová M., Titiš J., Krzystek J., Boča R. (2013). Inorg. Chem..

[cit13] Saber M. R., Dunbar K. R. (2014). Chem. Commun..

[cit14] Vaidya S., Upadhyay A., Singh S. K., Gupta T., Tewary S., Langley S. K., Walsh J. P., Murray K. S., Rajaraman G., Shanmugam M. (2015). Chem. Commun..

[cit15] Yang F., Zhou Q., Zhang Y., Zeng G., Li G., Shi Z., Wang B., Feng S. (2013). Chem. Commun..

[cit16] Zadrozny J. M., Liu J., Piro N. A., Chang C. J., Hill S., Long J. R. (2012). Chem. Commun..

[cit17] Zadrozny J. M., Long J. R. (2011). J. Am. Chem. Soc..

[cit18] Zadrozny J. M., Telser J., Long J. R. (2013). Polyhedron.

[cit19] Fataftah M. S., Zadrozny J. M., Rogers D. M., Freedman D. E. (2014). Inorg. Chem..

[cit20] Eichhöfer A., Lan Y., Mereacre V., Bodenstein T., Weigend F. (2014). Inorg. Chem..

[cit21] Ruamps R., Batchelor L. J., Guillot R., Zakhia G., Barra A. L., Wernsdorfer W., Guihery N., Mallah T. (2014). Chem. Sci..

[cit22] Jurca T., Farghal A., Lin P. H., Korobkov I., Murugesu M., Richeson D. S. (2011). J. Am. Chem. Soc..

[cit23] Habib F., Luca O. R., Vieru V., Shiddiq M., Korobkov I., Gorelsky S. I., Takase M. K., Chibotaru L. F., Hill S., Crabtree R. H., Murugesu M. (2013). Angew. Chem., Int. Ed. Engl..

[cit24] Vallejo J., Castro I., Ruiz-García R., Cano J., Julve M., Lloret F., De Munno G., Wernsdorfer W., Pardo E. (2012). J. Am. Chem. Soc..

[cit25] Chandrasekhar V., Dey A., Mota A. J., Colacio E. (2013). Inorg. Chem..

[cit26] Colacio E., Ruiz J., Ruiz E., Cremades E., Krzystek J., Carretta S., Cano J., Guidi T., Wernsdorfer W., Brechin E. K. (2013). Angew. Chem., Int. Ed. Engl..

[cit27] Wu D., Zhang X., Huang P., Huang W., Ruan M., Ouyang Z. W. (2013). Inorg. Chem..

[cit28] Gómez-Coca S., Urtizberea A., Cremades E., Alonso P. J., Camón A., Ruiz E., Luis F. (2014). Nat. Commun..

[cit29] Zhu Y. Y., Cui C., Zhang Y. Q., Jia J. H., Guo X., Gao C., Qian K., Jiang S. D., Wang B. W., Wang Z. M., Gao S. (2013). Chem. Sci..

[cit30] Herchel R., Váhovská L., Potočňák I., Trávníček Z. (2014). Inorg. Chem..

[cit31] Zhu Y.-Y., Zhang Y.-Q., Yin T.-T., Gao C., Wang B.-W., Gao S. (2015). Inorg. Chem..

[cit32] Novikov V. V., Pavlov A. A., Nelyubina Y. V., Boulon M.-E., Varzatskii O. A., Voloshin Y. Z., Winpenny R. E. P. (2015). J. Am. Chem. Soc..

[cit33] Plenk C., Krause J., Rentschler E. (2015). Eur. J. Inorg. Chem..

[cit34] Huang X. C., Zhou C., Shao D., Wang X. Y. (2014). Inorg. Chem..

[cit35] Chen L., Chen S.-Y., Sun Y.-C., Guo Y.-M., Yu L., Chen X.-T., Wang Z., Ouyang Z. W., Song Y., Xue Z.-L. (2015). Dalton Trans..

[cit36] Chen L., Wang J., Wei J. M., Wernsdorfer W., Chen X. T., Zhang Y. Q., Song Y., Xue Z. L. (2014). J. Am. Chem. Soc..

[cit37] Sun H.-L., Wang Z.-M., Gao S. (2010). Coord. Chem. Rev..

[cit38] Zhang W.-X., Ishikawa R., Breedlove B., Yamashita M. (2013). RSC Adv..

[cit39] Murrie M. (2010). Chem. Soc. Rev..

[cit40] Beedle C. C., Zhang Y.-Z., Holmes S. M., Hill S. (2013). Polyhedron.

[cit41] Li D., Parkin S., Wang G., Yee G. T., Prosvirin A. V., Holmes S. M. (2005). Inorg. Chem..

[cit42] Li D., Parkin S., Wang G., Yee G. T., Clérac R., Wernsdorfer W., Holmes S. M. (2006). J. Am. Chem. Soc..

[cit43] Zhang Y., Mallik U. P., Rath N., Yee G. T., Clérac R., Holmes S. M. (2010). Chem. Commun..

[cit44] Zhang Y.-Z., Mallik U. P., Clérac R., Rath N. P., Holmes S. M. (2011). Chem. Commun..

[cit45] Wang C.-F., Zuo J.-L., Bartlett B. M., Song Y., Long J. R., You X.-Z. (2006). J. Am. Chem. Soc..

[cit46] Li D., Clérac R., Parkin S., Wang G., Yee G. T., Holmes S. M. (2006). Inorg. Chem..

[cit47] Zhang Y.-Z., Mallik U. P., Rath N. P., Clérac R., Holmes S. M. (2011). Inorg. Chem..

[cit48] Schelter E. J., Prosvirin A. V., Dunbar K. R. (2004). J. Am. Chem. Soc..

[cit49] Schelter E. J., Karadas F., Avendano C., Prosvirin A. V., Wernsdorfer W., Dunbar K. R. (2007). J. Am. Chem. Soc..

[cit50] Saber M. R., Dunbar K. R. (2014). Chem. Commun..

[cit51] Wang S., Zuo J.-L., Gao S., Song Y., Zhou H.-C., Zhang Y.-Z., You X.-Z. (2004). J. Am. Chem. Soc..

[cit52] Hoshino N., Sekine Y., Nihei M., Oshio H. (2010). Chem. Commun..

[cit53] Mitsumoto K., Ui M., Nihei M., Nishikawa H., Oshio H. (2010). CrystEngComm.

[cit54] Novikov V. V., Pavlov A. A., Belov A. S., Vologzhanina A. V., Savitsky A., Voloshin Y. Z. (2014). J. Phys. Chem. Lett..

[cit55] Gruden-Pavlović M., Stepanović S., Perić M., Güell M., Swart M. (2014). Phys. Chem. Chem. Phys..

[cit56] Jesson J. P. (1966). J. Chem. Phys..

[cit57] Anderson P. A., Astley T., Hitchman M. A., Keene F. R., Moubaraki B., Murray K. S., Skelton B. W., Tiekink E. R. T., Toftlund H., White A. H. (2000). J. Chem. Soc., Dalton Trans..

[cit58] Schneider C. J., Moubaraki B., Cashion J. D., Turner D. R., Leita B. A., Batten S. R., Murray K. S. (2011). Dalton Trans..

[cit59] Goodman M. A., Nazarenko A. Y., Casavant B. J., Li Z., Brennessel W. W., DeMarco M. J., Long G., Goodman M. S. (2012). Inorg. Chem..

[cit60] Wanke R., Guedes da Silva M. F. C., Lancianesi S., Silva T. F. S., Martins L. M. D. R. S., Pettinari C., Pombeiro A. J. L. (2010). Inorg. Chem..

[cit61] Reger D. L., Elgin J. D., Foley E. A., Smith M. D., Grandjean F., Long G. J. (2009). Inorg. Chem..

[cit62] Moubaraki B., Leita B. A., Halder G. J., Batten S. R., Jensen P., Smith J. P., Cashion J. D., Kepert C. J., Letard J. F., Murray K. S. (2007). Dalton Trans..

[cit63] Reger D. L., Elgin J. D., Smith M. D., Grandjean F., Rebbouh L., Long G. J. (2004). Eur. J. Inorg. Chem..

[cit64] Reger D. L., Little C. A., Rheingold A. L., Lam M., Concolino T., Mohan A., Long G. J. (2000). Inorg. Chem..

[cit65] Kuzu I., Krummenacher I., Hewitt I. J., Lan Y., Mereacre V., Powell A. K., Höfer P., Harmer J., Breher F. (2009). Chem.–Eur. J..

[cit66] Reger D. L., Grattan T. C., Brown K. J., Little C. A., Lamba J. J. S., Rheingold A. L., Sommer R. D. (2000). J. Organomet. Chem..

[cit67] Neese F. (2012). WIREs Comput. Mol. Sci..

[cit68] Schaefer A., Huber C., Ahlrichs R. (1994). J. Chem. Phys..

[cit69] Weigend F. (2006). Phys. Chem. Chem. Phys..

[cit70] Llunell M., Casanova D., Cirera J., Alemany P. and Alvarez S., Shape program (version 2.0), Universitat de Barcelona, Barcelona, Spain, 2010.

[cit71] Alvarez S., Alemany P., Casanova D., Cirera J., Llunell M., Avnir D. (2005). Coord. Chem. Rev..

[cit72] Alvarez S. (2015). Chem. Rev..

[cit73] Chilton N. F., Anderson R. P., Turner L. D., Soncini A., Murray K. S. (2013). J. Comput. Chem..

[cit74] Abragam A. and Bleaney B., Electron Paramagnetic Resonance of Transition Ions, Oxford University Press, Oxford, 1970.

[cit75] Atanasov M., Ganyushin D., Pantazis D. A., Sivalingam K., Neese F. (2011). Inorg. Chem..

[cit76] Kramers H. A. (1930). Proc. R. Acad. Sci. Amsterdam.

[cit77] Van Vleck J. H. (1940). Phys. Rev..

[cit78] Ishikawa N., Sugita M., Wernsdorfer W. (2005). Angew. Chem., Int. Ed..

[cit79] Zadrozny J. M., Atanasov M., Bryan A. M., Lin C. Y., Rekken B. D., Power P. P., Neese F., Long J. R. (2013). Chem. Sci..

[cit80] Díaz-Torres R., Menelaou M., Roubeau O., Sorrenti A., Brandariz-de-Pedro G., Sañudo E. C., Teat S. J., Fraxedas J., Ruiz E., Aliaga-Alcalde N. (2016). Chem. Sci..

[cit81] Marriott K. E. R., Bhaskaran L., Wilson C., Medarde M., Ochsenbein S. T., Hill S., Murrie M. (2015). Chem. Sci..

[cit82] Rechkemmer Y., Breitgoff F. D., van der Meer M., Atanasov M., Hakl M., Orlita M., Neugebauer P., Neese F., Sarkar B., van Slageren J. (2016). Nat. Commun..

[cit83] Zhu Y.-Y., Zhu M.-S., Yin T.-T., Meng Y.-S., Wu Z.-Q., Zhang Y.-Q., Gao S. (2015). Inorg. Chem..

[cit84] Vallejo J., Fortea-Pérez F. R., Pardo E., Benmansour S., Castro I., Krzystek J., Armentano D., Cano J. (2016). Chem. Sci..

[cit85] Meng Y.-S., Mo Z., Wang B.-W., Zhang Y.-Q., Deng L., Gao S. (2015). Chem. Sci..

[cit86] Carl E., Demeshko S., Meyer F., Stalke D. (2015). Chem.–Eur. J..

[cit87] Miklovič J., Valigura D., Boča R., Titiš J. (2015). Dalton Trans..

[cit88] Orbach R. (1961). Proc. R. Soc. London, Ser. A.

