# Supplementary material for: Trigonal antiprismatic Co(ii) single molecule magnets with large uniaxial anisotropies: importance of Raman and tunneling mechanisms
Source: Chem Sci. 2016 Jun 17;7(10):6519–27. doi: 10.1039/c6sc02035f (PMC5125373; doi:10.1039/c6sc02035f)
Supplement: Supplementary file 1 [file SC-007-C6SC02035F-s001.pdf]

Electronic Supplementary Material (ESI) for Chemical Science.
This journal is © The Royal Society of Chemistry 2016

Supporting Information

Trigonal Antiprismatic Co(II) Single Molecule Magnets with Large Uniaxial Anisotropies: Importance of Raman and Tunneling Mechanisms

Y.-Z. Zhang, S. Gómez-Coca, A. J. Brown, M. R. Saber, X. Zhang, K. R. Dunbar[a]

[a]*Department of Chemistry, Texas A & M University, College Station, TX 77842-3012 (USA). Fax: (+1) 979-845-7177*

Table of Contents

Single Crystal X-ray Diffraction Studies.
Figure S1. Packing view of **1**.
Figure S2. Packing view of **2**.
Static Magnetic Measurements
Figure S3. M vs. H/T plots at 2, 4, 6 K for **1** and **2**.
Dynamic Magnetic Measurements. Compound 1.
Figure S4. Variable-frequency ac magnetic susceptibility data for **1** collected at 1.8 K, an ac field of 5 Oe and dc fields from 0 to 5 kOe.
Figure S5. Cole-Cole plots of **1** at 1.8 K, an ac field of 5 Oe and dc fields from 0.25 to 5 kOe.
Table S1. Cole-Cole fit values of **1** at 1.8 K, an ac field of 5 Oe and dc fields from 0.25 to 5 kOe.
Figure S6. Variable-temperature ac magnetic susceptibility data for **1** collected at temperatures from 2 to 12 K, an ac field of 5 Oe and a dc field of 500 Oe.
Figure S7. Variable-frequency ac magnetic susceptibility data for **1** collected at 5.0 K, an ac field of 5 Oe and dc fields from 0.125 to 10 kOe.
Figure S8. Cole-Cole diagrams of **1** at 5.0 K, an ac field of 5 Oe and dc fields from 0.125 to 10 kOe.
Table S2. Cole-Cole fit values of **1** at 5.0 K, an ac field of 5 Oe and dc fields from 0.125 to 10 kOe.
Figure S9. Dependence of τ and τ^{-1} with the applied DC fields for complex **1** at 5 K.
Figure S10. Variable-frequency ac magnetic susceptibility data for **1** collected at temperatures from 2 to 8 K, an ac field of 5 Oe and a dc field of 500 Oe.
Figure S11. Cole-Cole plots of **1** from 2.0 to 8.0 K, an ac field of 5 Oe and a dc field of 500 Oe.
Table S3. Cole-Cole fit values of **1** from 2.0 to 8.0 K, an ac field of 5 Oe and a dc field of 500 Oe.
Figure S12. Cole-Cole diagrams of **1** from 2.0 to 8.0 K, an ac field of 5 Oe and a dc field of 3 kOe.
Table S4. Cole-Cole fit values of **1** from 2.0 to 8.0 K, an ac field of 5 Oe and a dc field of 3 kOe.
Dynamic Magnetic Measurements. Compound 2.
Figure S13. Variable-frequency ac magnetic susceptibility data for **2** collected at 1.8 K, an ac field of 5 Oe and dc fields from 0 to 1 kOe.
Figure S14. Variable-temperature ac magnetic susceptibility data for **2** collected at temperatures from 2 to 15 K, an ac field of 5 Oe and a dc field of 500 Oe.

Figure S15. Variable-frequency ac magnetic susceptibility data for **2** collected at 6.0 K, an ac field of 5 Oe and dc fields from 0 to 10 kOe.

Figure S16. Cole-Cole diagrams of **2** at 6.0 K, an ac field of 5 Oe and dc fields from 0.15 to 10 kOe.

Table S5. Cole-Cole fit values of **2** at 6.0 K, an ac field of 5 Oe and dc fields from 0.15 to 10 kOe.

Figure S17. Dependence of τ and τ^{-1} with the applied DC fields for complex **2** at 6.0 K.

Figure S18. Variable-frequency ac magnetic susceptibility data for **2** collected at temperatures from 2 to 9 K, an ac field of 5 Oe and a dc field of 300 Oe.

Figure S19. Cole-Cole diagrams of **2** from 2.0 to 11.0 K, an ac field of 5 Oe and a dc field of 300 Oe.

Table S6. Cole-Cole fit values of **2** from 2.0 to 11.0 K, an ac field of 5 Oe and a dc field of 300 Oe.

Figure S20. Variable-frequency ac magnetic susceptibility data for **2** collected at temperatures from 2 to 9.5 K, an ac field of 5 Oe and a dc field of 500 Oe.

Figure S21. Cole-Cole plots of **2** from 2.0 to 12.0 K, an ac field of 5 Oe and a dc field of 500 Oe.

Table S7. Cole-Cole fit values of **2** from 2.0 to 12.0 K, an ac field of 5 Oe and a dc field of 500 Oe.

Figure S22. Cole-Cole plots of **2** from 2.0 to 9.5 K, an ac field of 5 Oe and a dc field of 1500 Oe.

Table S8. Cole-Cole fit values of **2** from 2.0 to 9.5 K, an ac field of 5 Oe and a dc field of 1500 Oe.

Analysis of dynamic magnetic measurements.

Figure S23. Arrhenius plots of ln(τ) vs T^{-1} for 1 (left) and 2 (right) under different dc fields.

Figure S24. τ^{-1} vs temperature for **1** (left) and **2** (right) at different applied dc fields. The solid line is the best fit obtained using equation S2.

Table S9. Parameters obtained for the fit of the dependence of τ with temperature at different applied dc fields using equation S2 for **1** and **2**.

Figure S25. τ^{-1} vs temperature for **1** at 500 Oe (left) and 3000 Oe (right) with the contribution of Orbach, Raman and tunneling processes.

Figure S26. τ^{-1} vs temperature for **1** (left) and **2** (right) at different applied dc fields. The solid line is the best fit obtained using equation S3.

Table S10. Parameters obtained for the fit of the dependence of τ with temperature at different applied dc fields using equation S3 for **1** and **2**.

Computational Details

Table S11. Relative energy (E) in cm^{-1} and Boltzman population at 300 K (BP) of the twelve lowest states computed at CASSCF and NEVPT2 level.

Table S12. D and E/D values at CASSCF and NEVPT2 level.

Table S13. Energy of the first five excited states (cm^{-1}) and its main contributions to the D and E values in cm^{-1} at CAS(7,5) NEVPT2 level.

References

Single Crystal X-ray Diffraction Studies

Single crystal X-ray data for **1** and **2** were collected at 110 K on a Bruker APEX diffractometer equipped with a CCD detector. The data sets were recorded as ϖ-scans at 1.0° step widths. Integration was performed with the Bruker SAINT software package[S1] and absorption corrections were empirically applied using SADABS.[S2] The crystal structures were refined using the SHELX suite of programs.[S3] Images of the crystal structures were rendered using the visualization software DIAMOND.[S4] All of the structures were solved by direct methods and all non-hydrogen atoms were located by alternating cycles of least squares refinements and difference Fourier maps. All hydrogen atoms were placed at calculated positions except for some water molecules whose hydrogen atoms were located by difference Fourier maps. The bond distances in disordered solvent molecules were restrained to chemically meaningful values. Anisotropic thermal parameters were added for all non-hydrogen atoms. A summary of pertinent information relating to unit cell parameters, data collection, and refinement statistics is provided in Table 1. CCDC 1422285 (**1**) and 1422286 (**2**) contains the supplementary crystallographic data for this paper. These data can be obtained free of charge from the Cambridge Crystallographic Data Centre via www.ccdc.cam.ac.uk/data_request/cif.

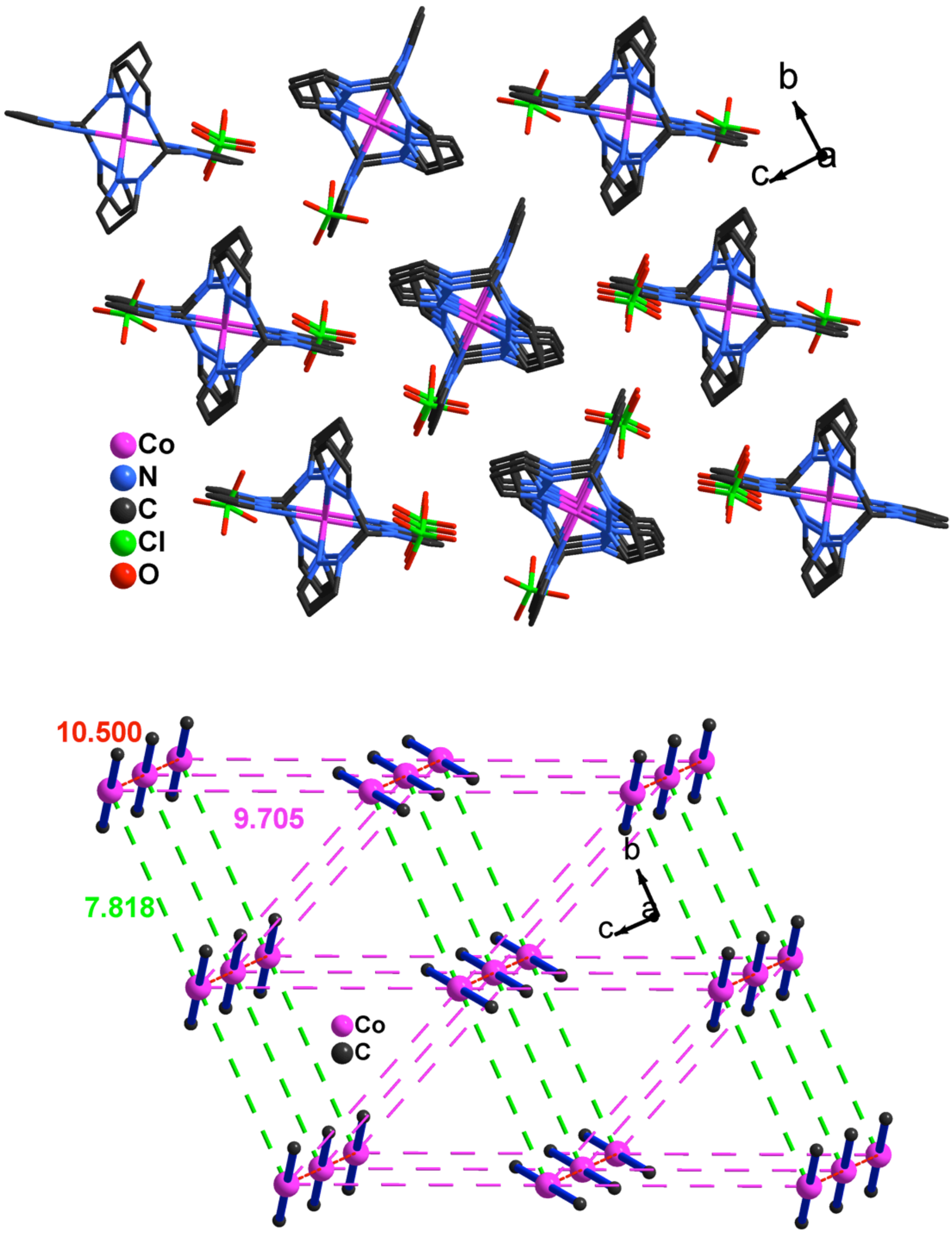

Figure S1. (top) Packing view of **1**, where the neighboring separations between Co(II) centers are 7.818(6), 9.705(42), and 10.500(8) Å; (bottom) Two orientations (C_3 axis) with an angle of 40.8° in **1**.

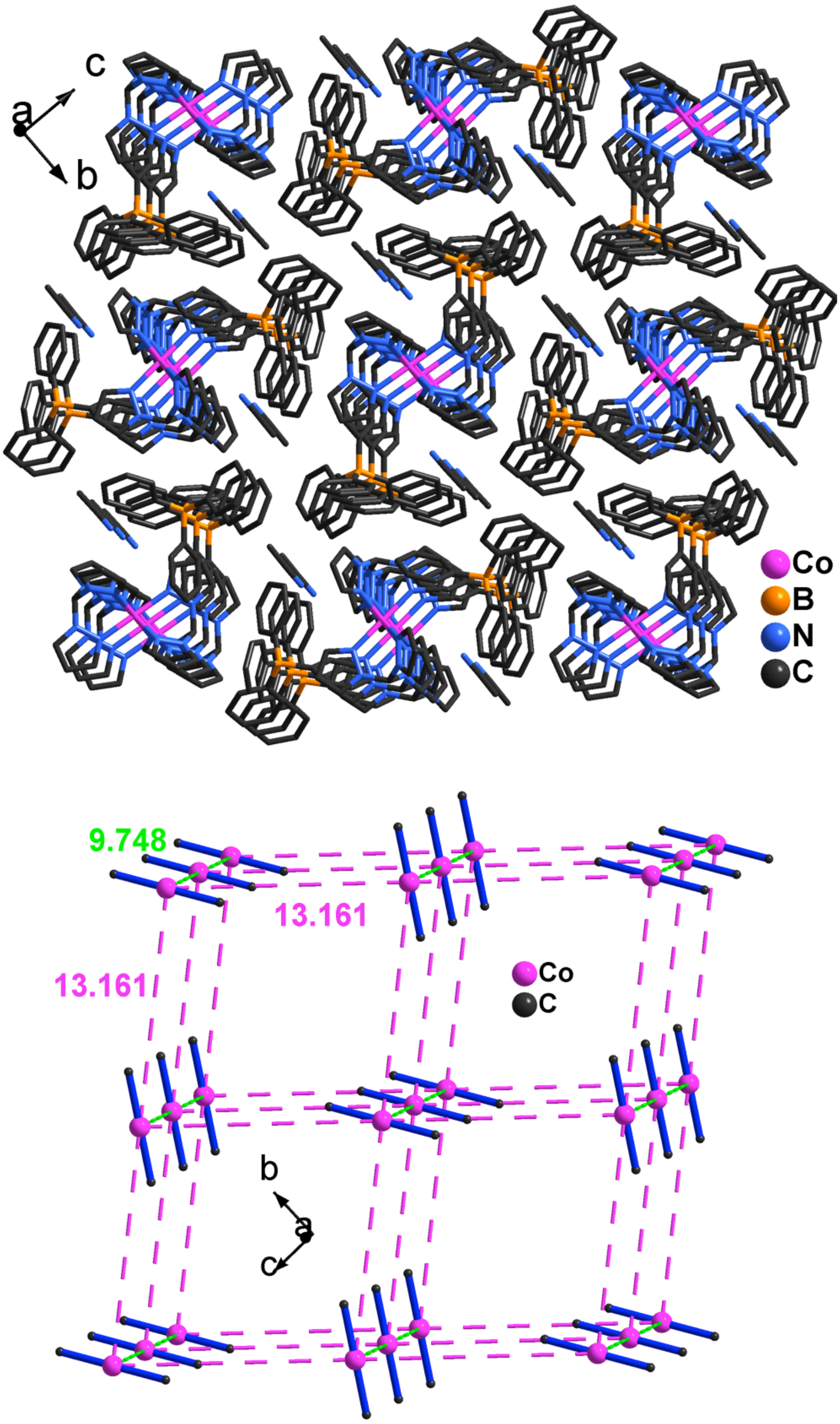

Figure S2. (top) Packing view of **2**, where the neighboring separations between Co(II) centers are 9.748(1), 13.161(3), and 13.161(3) Å; (bottom) Two orientations (C_3 axis) with an angle of 69.8° in **2**.

Static Magnetic Measurements

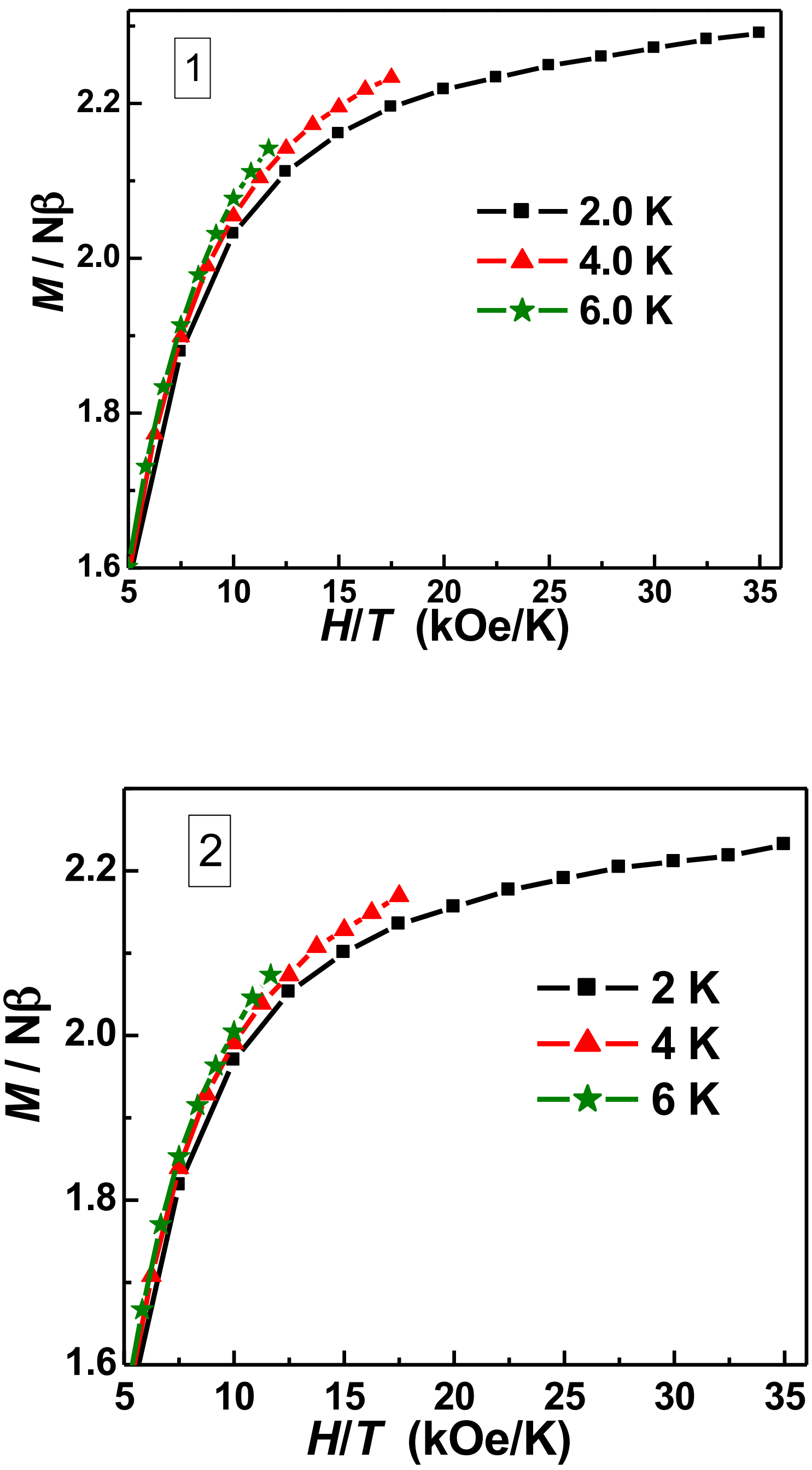

Figure S3. *M* vs. *H*/*T* plots at 2, 4, 6 K for **1** and **2**. Solid lines are a guide for the eyes.

Dynamic Magnetic Measurements. Compound 1

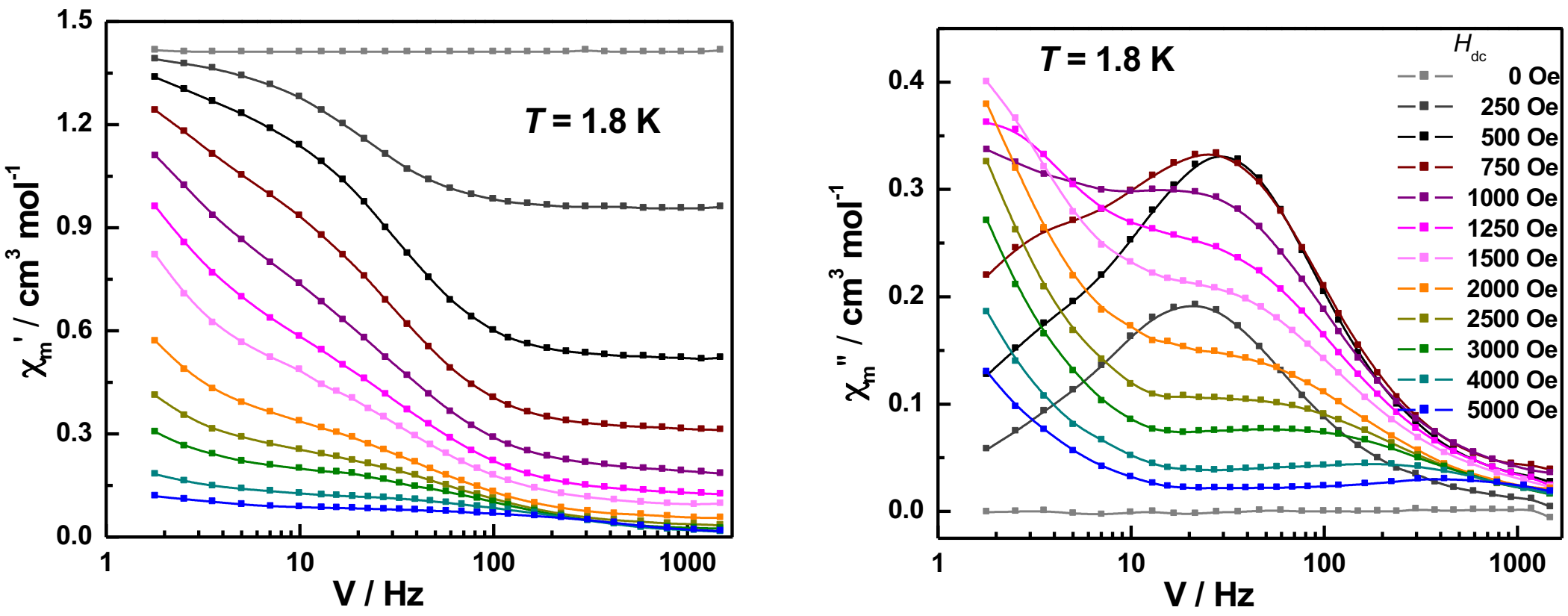

Figure S4. Variable-frequency in-phase (χ_m', up) and out-of-phase (χ_m'', down) components of the ac magnetic susceptibility data for **1**, collected at 1.8 K with an ac field of 5 Oe and 0, 250, 500, 750, 1000, 1250, 1500, 2000, 2500, 3000, 4000, and 5000 Oe dc applied fields, respectively. Solid lines are guides for the eye.

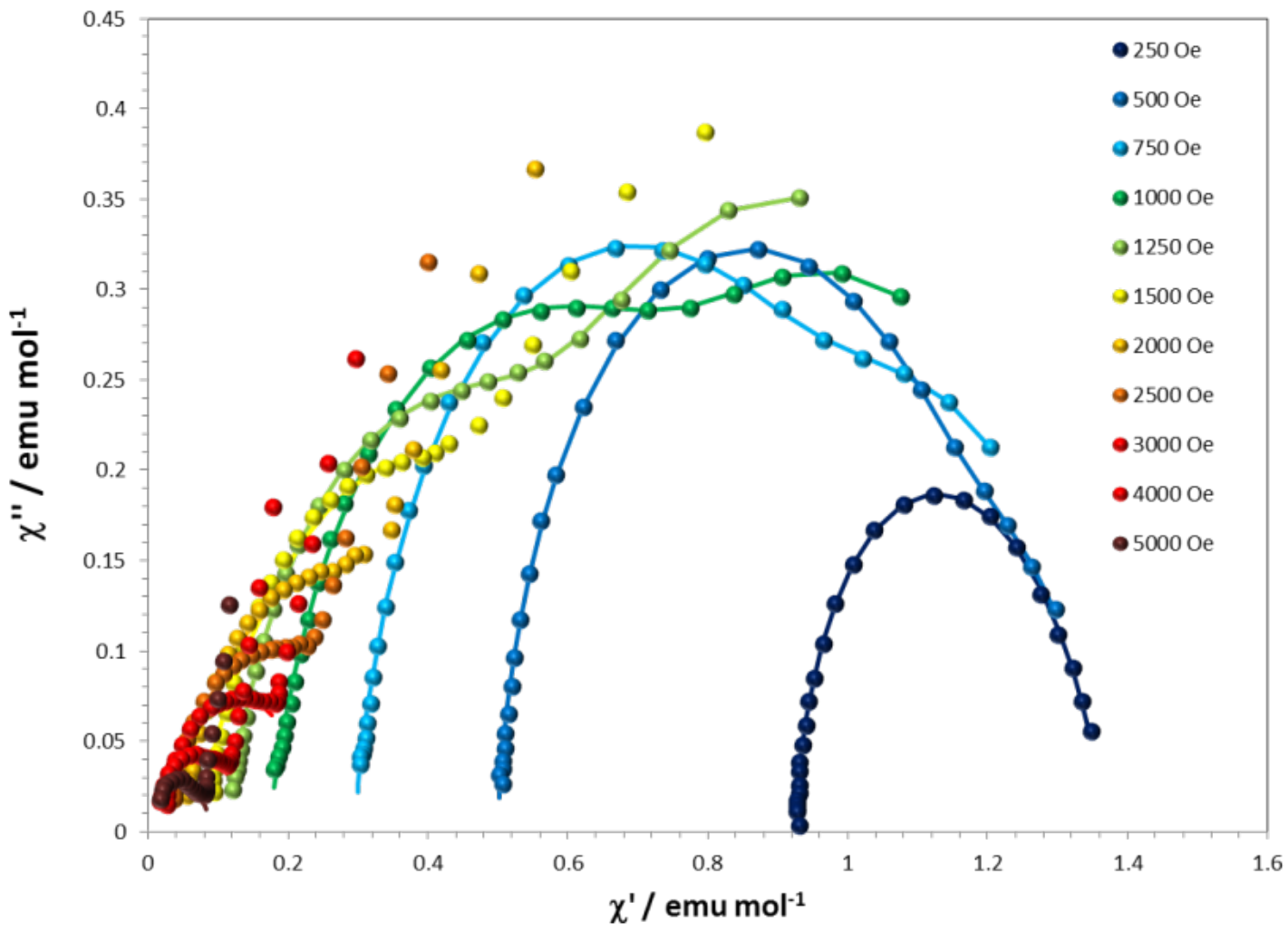

Figure S5. Cole-Cole diagrams of **1** at 1.8 K with an applied dc field from 0.25 to 5 kOe and ac field of 5 Oe. The solid lines are least-square fittings of the data to a distribution of single relaxation processes with a generalized Debye model.

Table S1. Cole-Cole fit values of **1** at 1.8 K with an applied dc field of 0.25 to 5 kOe and ac field of 5 Oe. Values in red have an α value of ~ 0.3 indicating a large distribution of relaxation times.

Field (Oe)	χ_s (cm^3 mol^{-1})	χ_{t1} (cm^3 mol^{-1})	τ1 (s)	α1	χ_{t2} (cm^3 mol^{-1})	τ2 (s)	α2	Residual
250	0.92400	0.25800	0.00660	0.00000	0.18600	0.01540	0.21000	0.00011
500	0.50000	0.66300	0.00503	0.06180	0.19900	0.05320	0.07950	0.00035
750	0.29500	0.67400	0.00516	0.10700	0.38500	0.06270	0.08140	0.00086
1000	0.17300	0.63400	0.00546	0.17300	0.52600	0.07850	0.05830	0.00041
1250	0.11400	0.48900	0.00468	0.17200	0.73600	0.09730	0.10300	0.00011
1500	0.06660	0.75100	0.00574	0.30100				0.00365
2000	0.03320	0.51600	0.00463	0.31400				0.00251
2500	0.01620	0.35200	0.00293	0.31600				0.00188
3000	0.00741	0.25100	0.00179	0.30900				0.00152
4000	0.00000	0.14300	0.00078	0.30600				0.00079
5000	0.00000	0.09140	0.00038	0.29600				0.00050

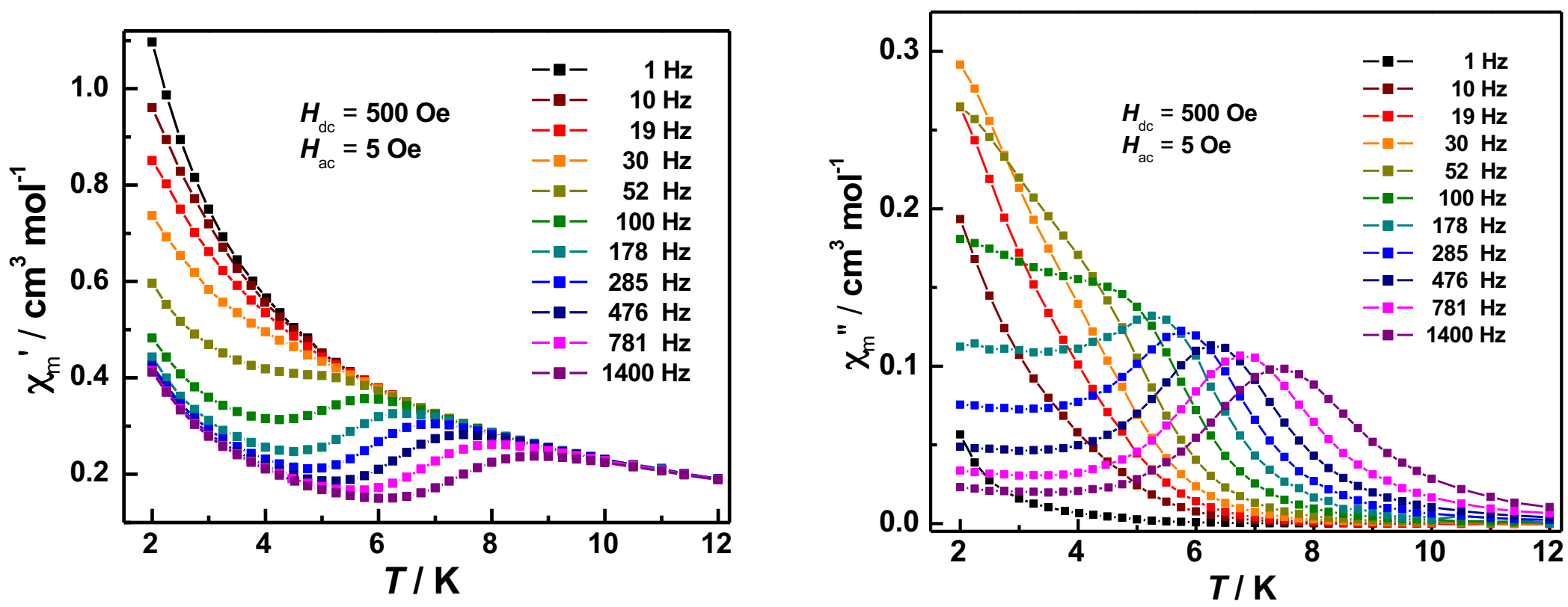

Figure S6. Variable-temperature in-phase (χ_m', top) and out-of-phase (χ_m'', bottom) components of the ac magnetic susceptibility data for **1**, collected at temperatures from 2 to 12 K with an ac filed of 5 Oe and 500 Oe dc applied fields. Solid lines are guides for the eye.

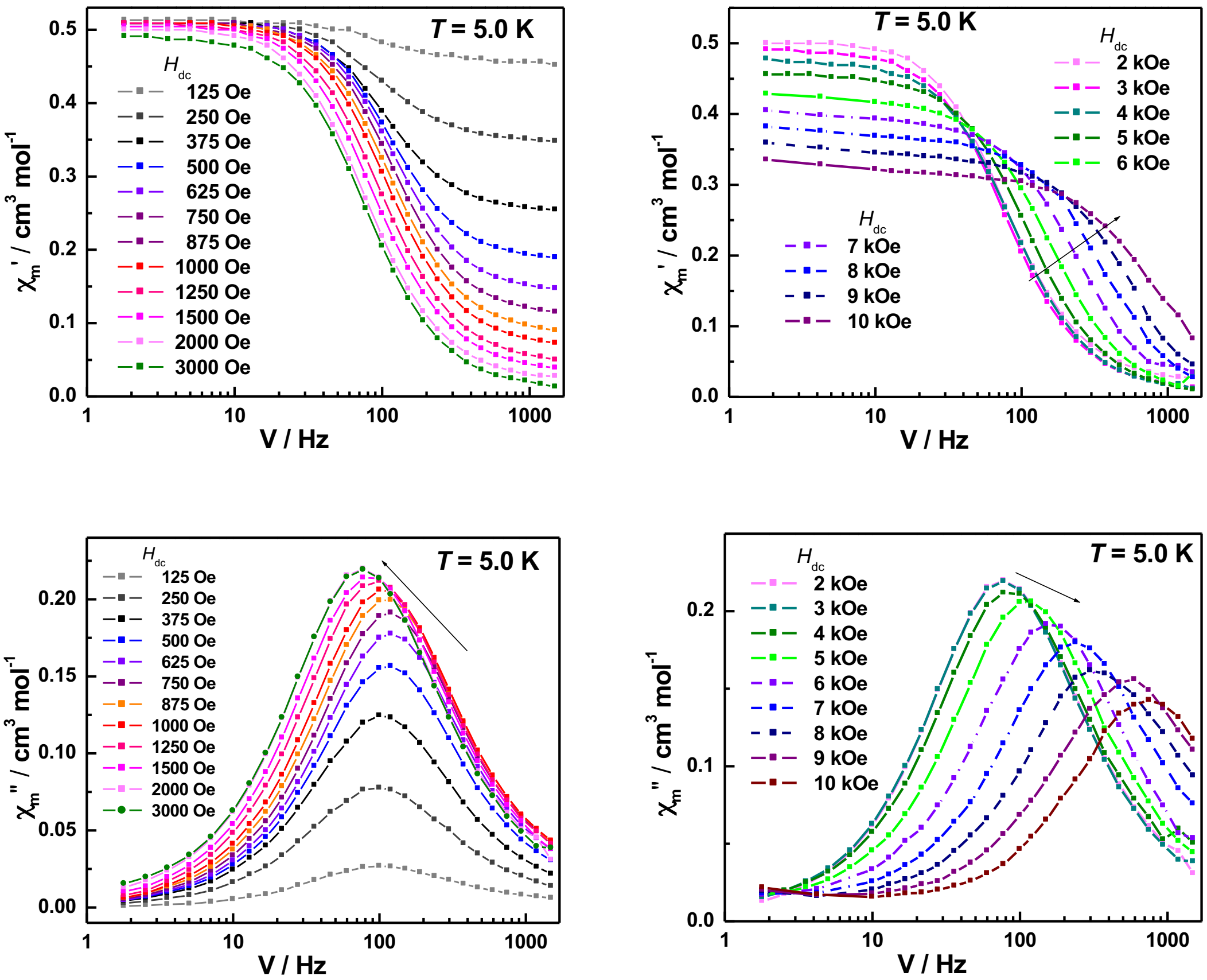

Figure S7. Variable-frequency in-phase (χ_m', top) and out-of-phase (χ_m'', bottom) components of the ac magnetic susceptibility data for **1**, collected at 5.0 K with an ac filed of 5 Oe and a dc applied field between 0.125 and 10 kOe, respectively. Solid lines are guides for the eye.

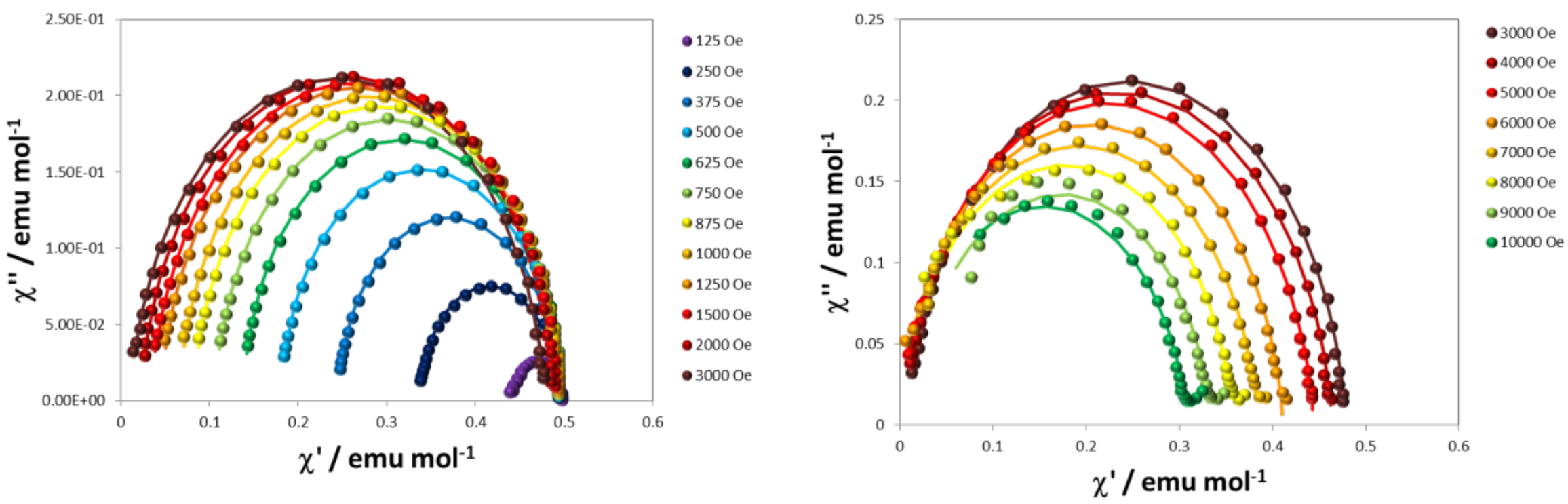

Figure S8. Cole-Cole plots of **1** at 5.0 K with an applied dc field of 0.125 to 10 kOe and ac field of 5 Oe. The solid lines are least-square fittings of the data to a distribution of single relaxation processes with a generalized Debye model.

Table S2. Cole-Cole fit values of **1** at 5.0 K with an applied dc field from 0.125 to 10 kOe and ac field of 5 Oe.

Field (Oe)	χ_s (cm^3 mol^{-1})	χ_t (cm^3 mol^{-1})	τ (s)	α	R
125	0.44000	0.49700	0.00152	0.06110	0.000070
250	0.33700	0.49500	0.00156	0.03000	0.000077
375	0.24500	0.49500	0.00149	0.02520	0.000070
500	0.18100	0.49500	0.00135	0.02080	0.000100
625	0.13800	0.49600	0.00130	0.02640	0.000155
750	0.10700	0.49600	0.00132	0.03410	0.000222
875	0.08290	0.49600	0.00137	0.04260	0.000231
1000	0.06570	0.49700	0.00145	0.04820	0.000293
1250	0.04450	0.49600	0.00160	0.05880	0.000294
1500	0.03380	0.49400	0.00175	0.06220	0.000348
2000	0.02220	0.49000	0.00197	0.06000	0.000241
3000	0.01070	0.47900	0.00199	0.06280	0.000168
4000	0.00573	0.46400	0.00179	0.06990	0.000257
5000	0.00212	0.44400	0.00139	0.06860	0.000276
6000	0.00000	0.41100	0.00099	0.06530	0.000268
7000	0.00000	0.38400	0.00067	0.06960	0.000435
8000	0.00000	0.35800	0.00045	0.06990	0.000366
9000	0.01110	0.33400	0.00031	0.08190	0.000835
10000	0.00000	0.30800	0.00021	0.08610	0.000324

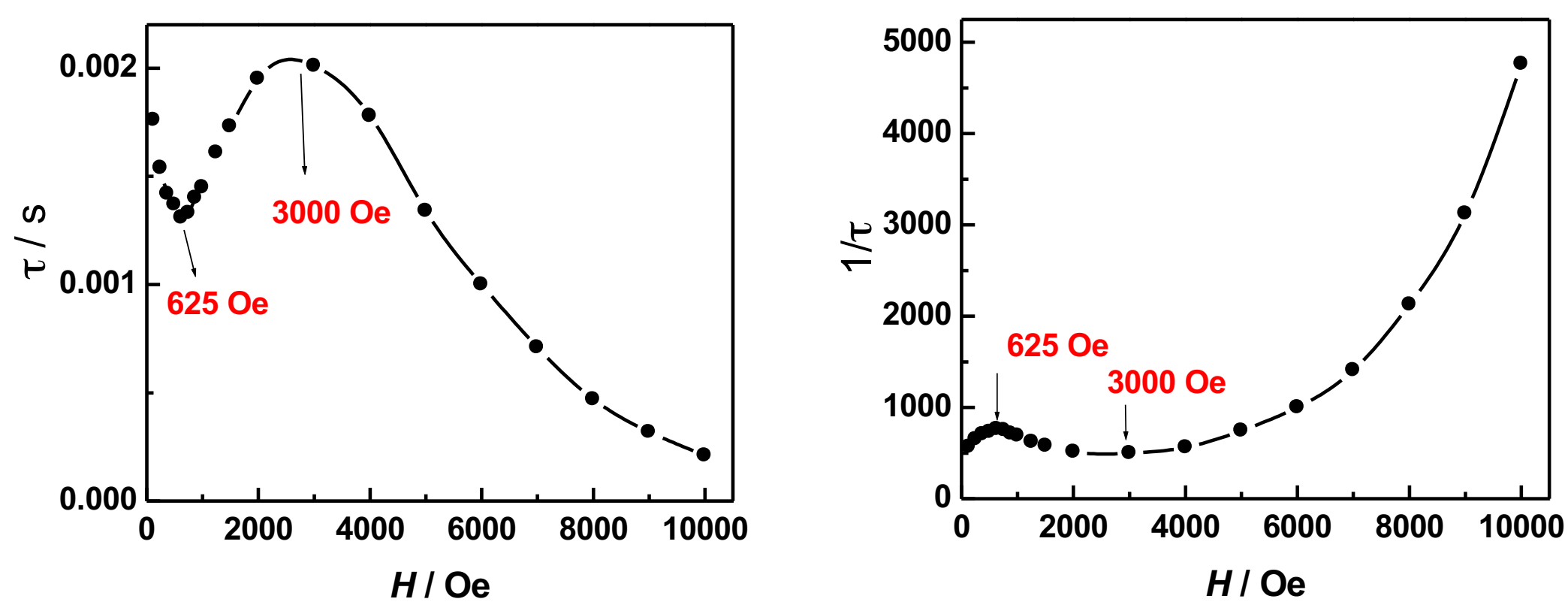

Figure S9. Dependence of τ (left) and τ^{-1} (right) with the applied DC fields for complex **1** at 5 K. Solid lines are guides for the eye.

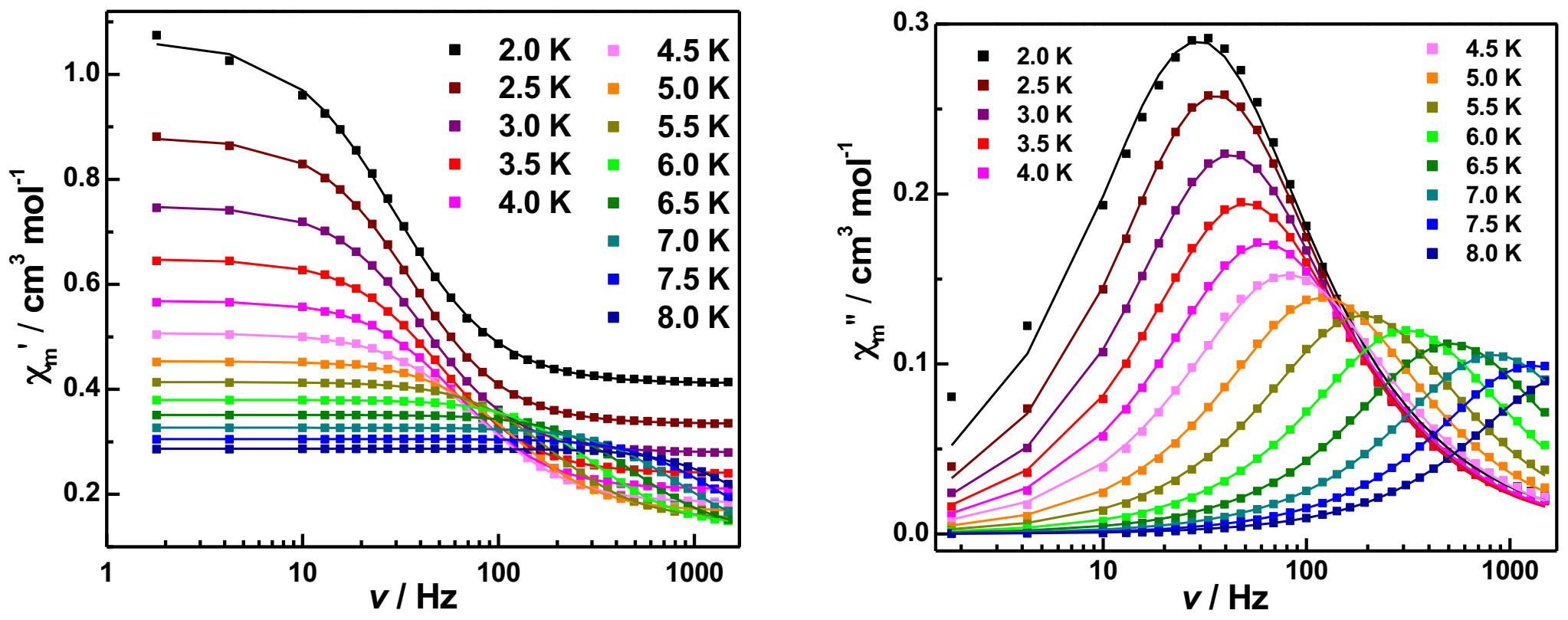

Figure S10. Variable-frequency in-phase (χ_m', top) and out-of-phase (χ_m'', bottom) components of the ac magnetic susceptibility data for **1**, collected at temperatures from 2 to 8 K with an ac field of 5 Oe and 500 Oe dc applied fields.

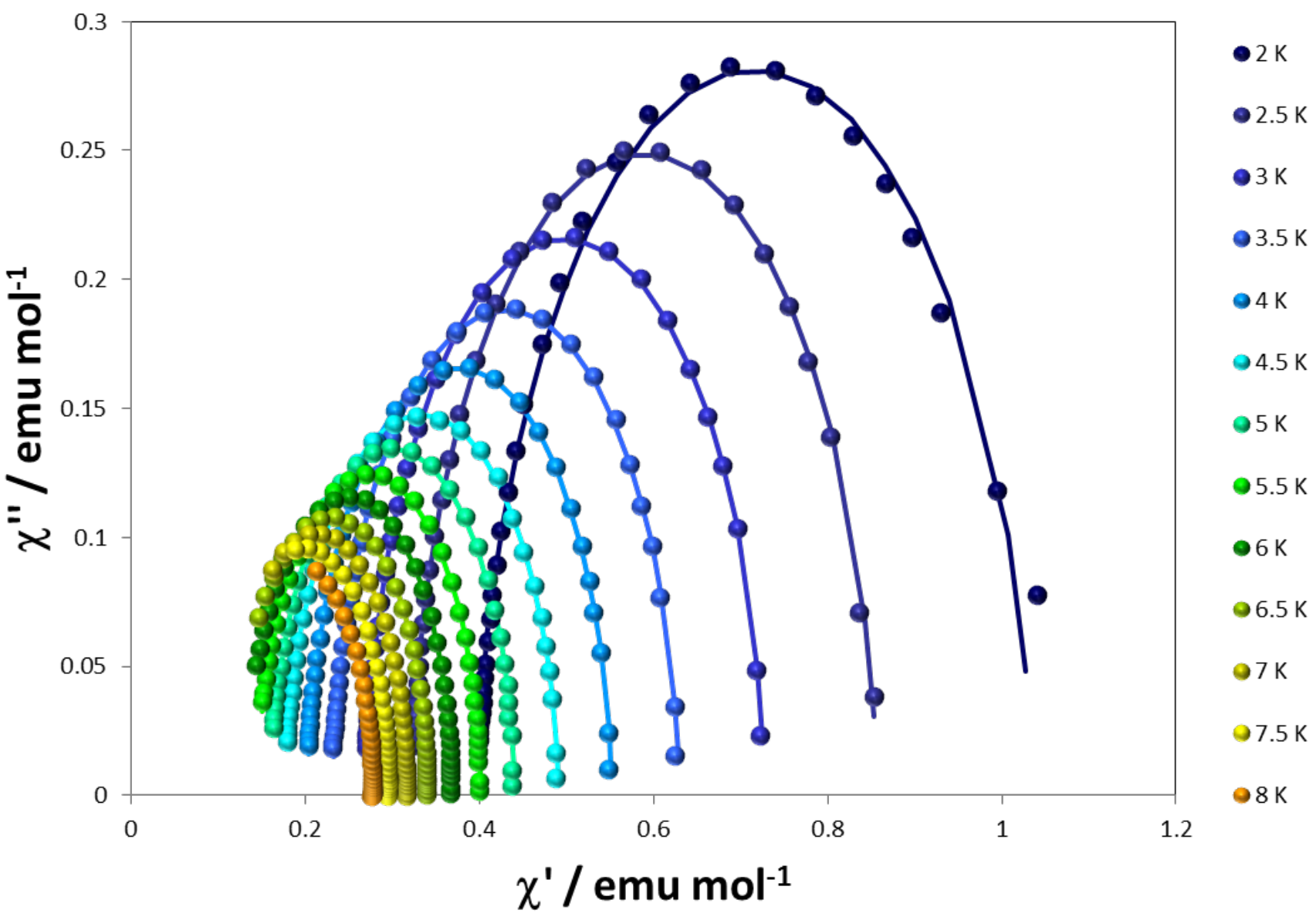

Figure S11. Cole-Cole diagrams of **1** from 2.0 to 8.0 K with an applied dc field of 500 Oe and an ac field of 5 Oe. The solid lines are least-square fittings of the data to a distribution of single relaxation processes with a generalized Debye model.

Table S3. Cole-Cole fit values of **1** from 2.0 to 8.0 K, ac field of 5 Oe and dc field of 500 Oe.

T / K	χ_s (cm^3 mol^{-1})	χ_t (cm^3 mol^{-1})	τ (s)	α	R
2.0	0.39400	1.04000	0.00542	0.08480	0.0022900
2.5	0.32100	0.85700	0.00444	0.04600	0.0003020
3.0	0.26900	0.72800	0.00376	0.03830	0.0001330
3.5	0.23100	0.63000	0.00315	0.03520	0.0001030
4.0	0.20200	0.55200	0.00257	0.03350	0.0000897
4.5	0.17700	0.49100	0.00199	0.03770	0.0001070
5.0	0.16100	0.44000	0.00133	0.02380	0.0000690
5.5	0.14600	0.40100	0.00085	0.01800	0.0000509
6.0	0.13200	0.36900	0.00051	0.01420	0.0000412
6.5	0.12100	0.34100	0.00031	0.00947	0.0000267
7.0	0.11100	0.31700	0.00019	0.00571	0.0000147
7.5	0.10300	0.29600	0.00012	0.00198	0.0000123
8.0	0.09530	0.27800	0.00008	0.00275	0.0000088

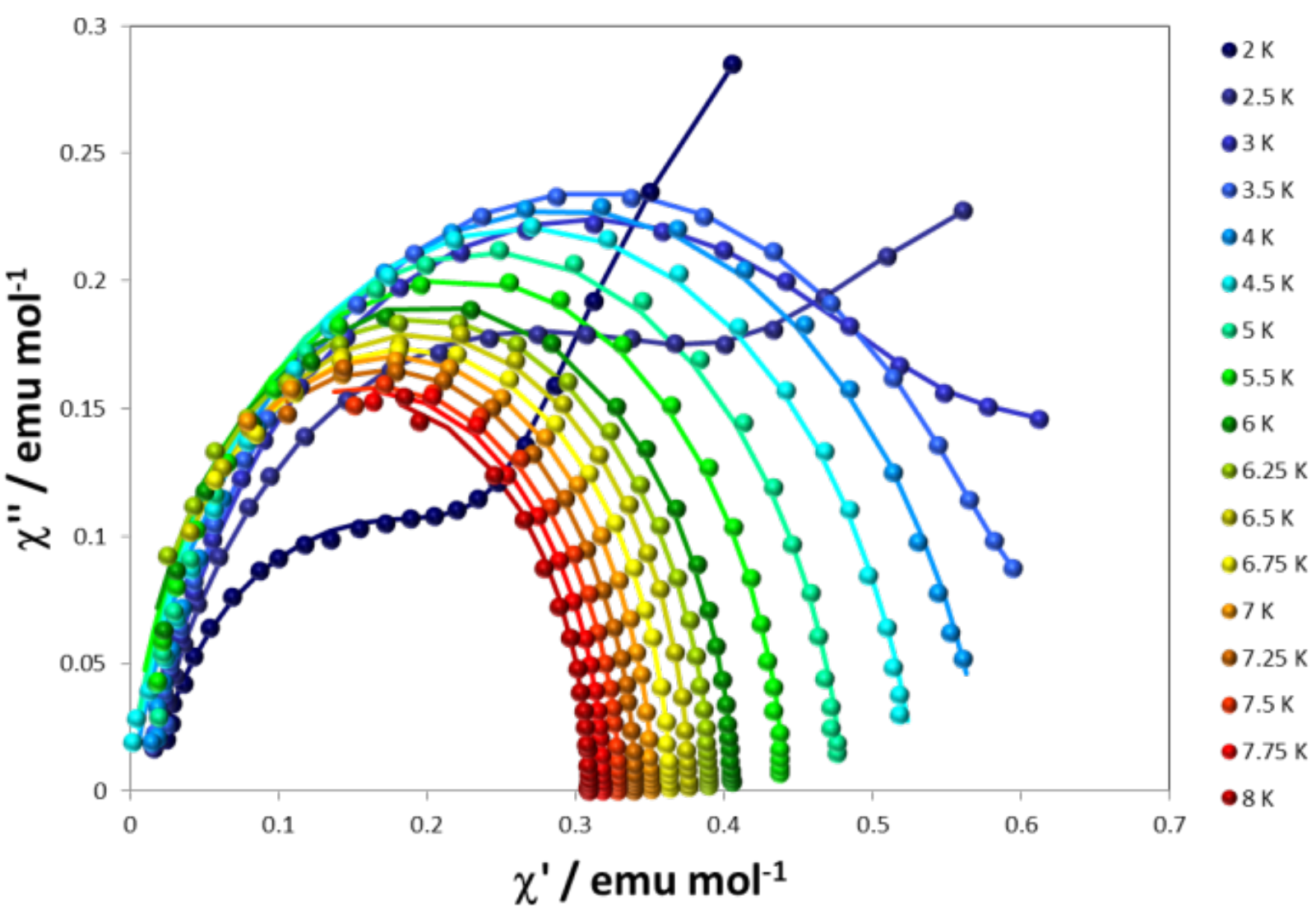

Figure S12. Cole-Cole diagrams of **1** from 2.0 to 8.0 K with an applied dc field of 3 kOe and an ac field of 5 Oe. The solid lines are least-square fittings of the data to a distribution of single relaxation processes (≥ 4.0 K) with a generalized Debye model.

Table S4. Cole-Cole fit values of **1** from 2.0 to 8.0 K, ac field of 5 Oe and dc field of 3 kOe.

T / K	χ_s (cm^3 mol^{-1})	χ_{t1} (cm^3 mol^{-1})	$\tau 1$ (s)	$\alpha 1$	χ_{t2} (cm^3 mol^{-1})	$\tau 2$ (s)	$\alpha 2$	Residual
2.0	0.01200	0.24100	0.00269	0.21200	0.97000	0.28100	0.13200	0.000071
2.5	0.01070	0.39300	0.00474	0.18000	0.62100	0.19700	0.19700	0.000064
3.0	0.01030	0.53700	0.00627	0.16500	0.26900	0.16300	0.17100	0.000060
3.5	0.01080	0.59000	0.00628	0.15300	0.14200	0.44300	0.14700	0.000178

T / K	χ_s (cm^3 mol^{-1})	χ_t (cm^3 mol^{-1})	τ (s)	α	Residual
4.0	0.00000	0.57800	0.00493	0.15000	0.000759
4.5	0.00000	0.53000	0.00330	0.11600	0.000578
5.0	0.00000	0.48100	0.00195	0.08200	0.000808
5.5	0.00000	0.44100	0.00109	0.05860	0.000500
6.0	0.00000	0.40600	0.00061	0.03860	0.000296
6.25	0.00007	0.39100	0.00045	0.03370	0.000200
6.5	0.00677	0.37700	0.00035	0.02080	0.000100
6.75	0.00675	0.36400	0.00027	0.01680	0.000055
7.0	0.00000	0.35200	0.00021	0.01940	0.000066
7.25	0.00000	0.34000	0.00016	0.01770	0.000081
7.5	0.00000	0.33000	0.00013	0.02450	0.000325
7.75	0.00000	0.31900	0.00010	0.00796	0.000081
8.0	0.00000	0.30900	0.00009	0.00000	0.000498

Dynamic Magnetic Measurements. Compound 2

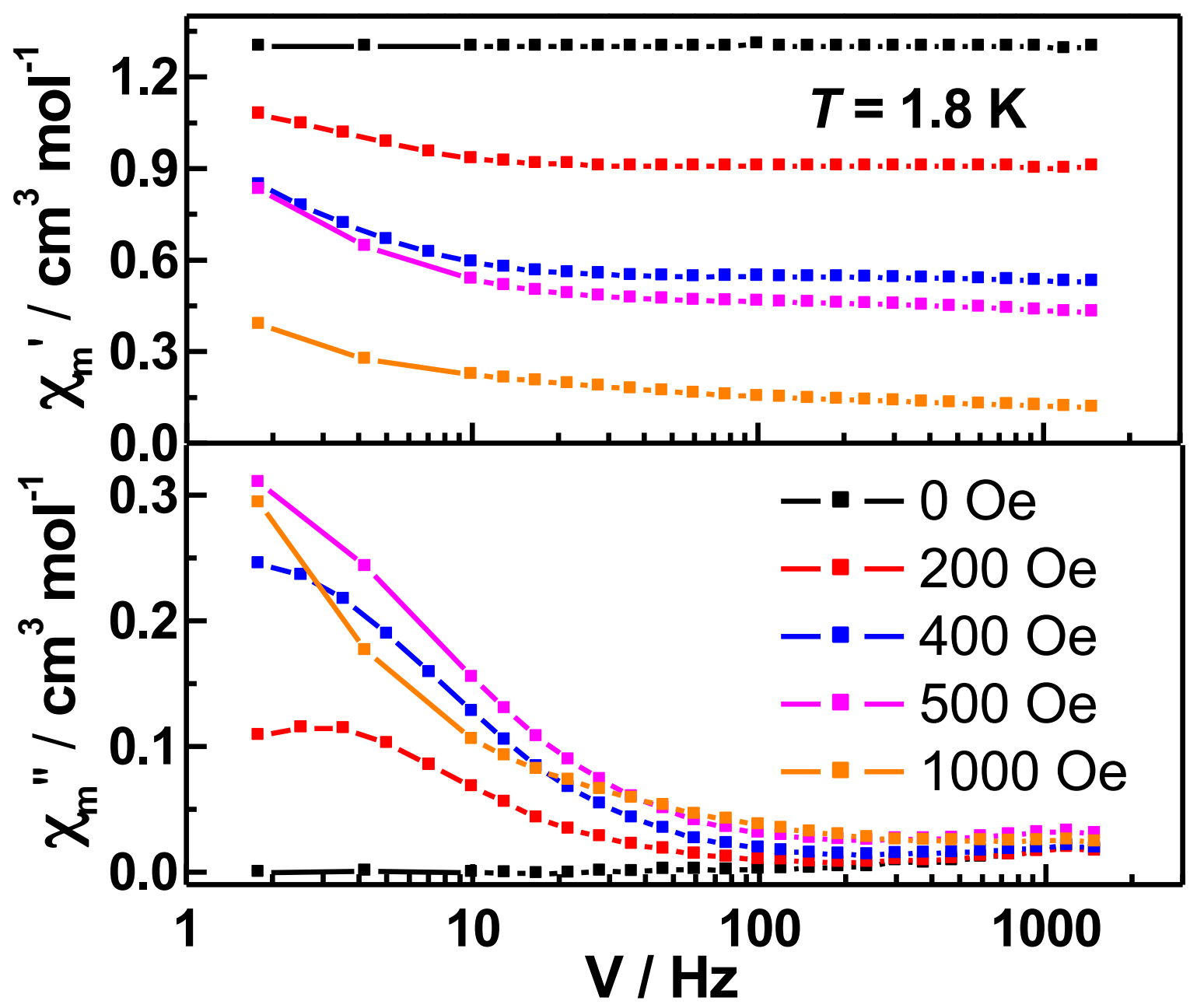

Figure S13. Variable-frequency in-phase (χ_m') and out-of-phase (χ_m'') components of the ac magnetic susceptibility data for **2**, collected at 1.8 K with an ac field of 5 Oe and different dc applied fields, respectively. Solid lines are guides for the eye.

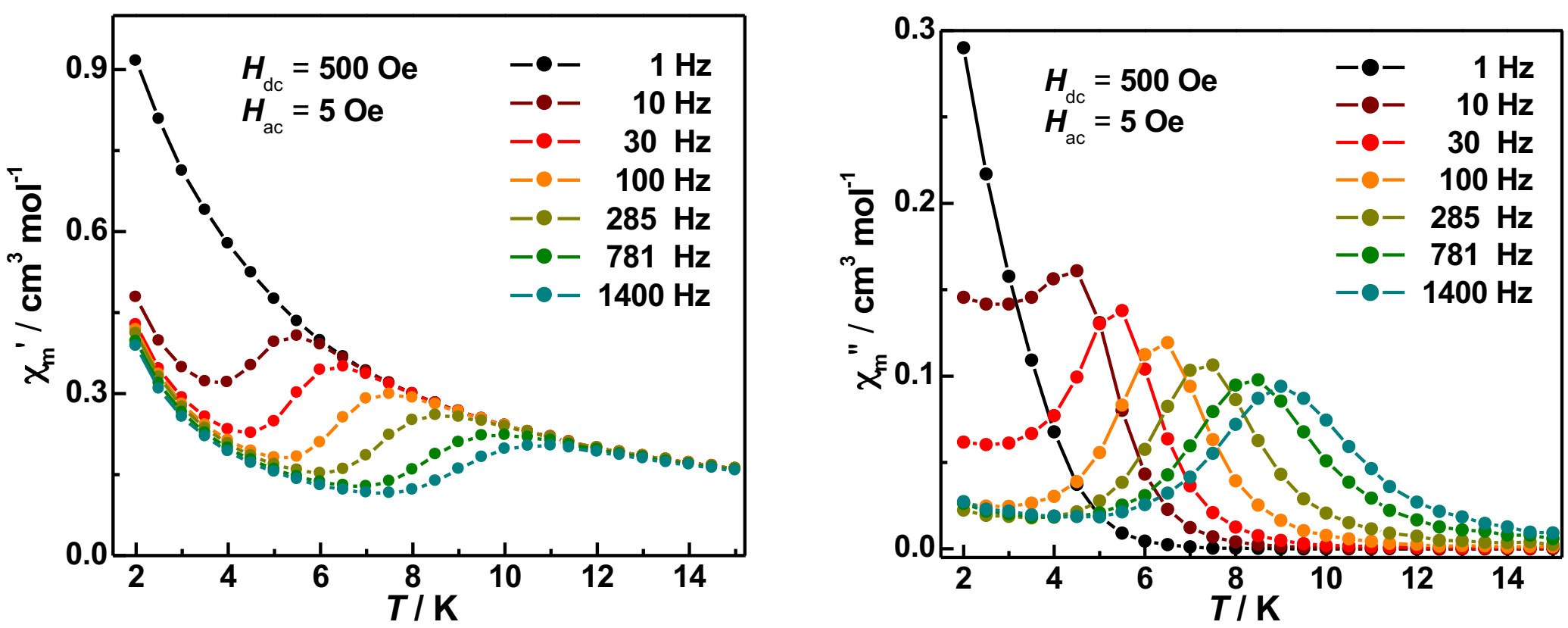

Figure S14. Variable-temperature in-phase (χ_m', top) and out-of-phase (χ_m'', bottom) components of the ac magnetic susceptibility data for **2**, collected at temperatures from 2 to 15 K with an ac field of 5 Oe and 500 Oe dc applied fields. Solid lines are guides for the eye.

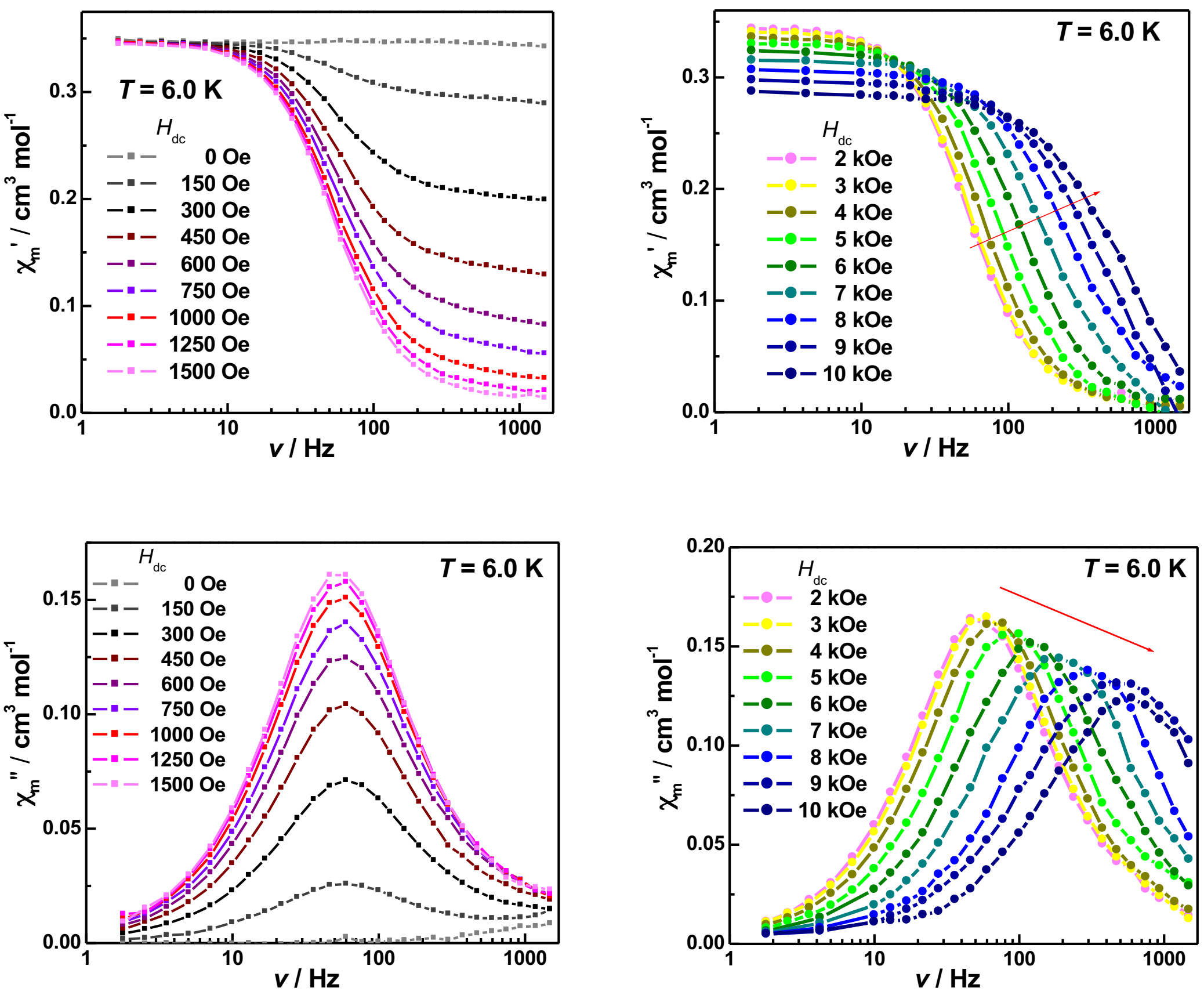

Figure S15. Variable-frequency in-phase (χ_m', top) and out-of-phase (χ_m'', bottom) components of the ac magnetic susceptibility data for **2**, collected at a temperature of 6.0 K with an ac field of 5 Oe and a dc applied field between 0 and 10 kOe, respectively. Solid lines are guides for the eye.

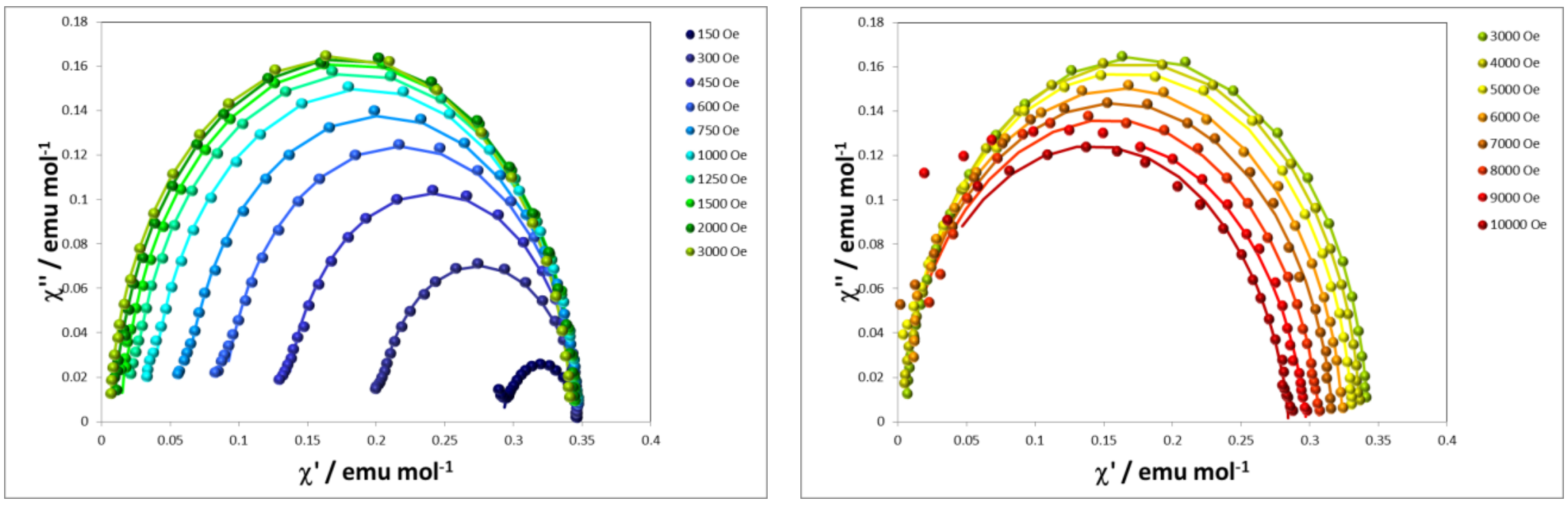

Figure S16. Cole-Cole diagrams of **2** at 6.0 K with an applied dc field from 0.15 to 10 kOe and ac field of 5 Oe. The solid lines are least-square fittings of the data to a distribution of single relaxation processes with a generalized Debye model.

Table S5. Cole-Cole fit values of **2** at 6.0 K, ac field of 5 Oe and dc fields from 0.15 to 10 kOe.

Field / Oe	χ_s (cm^3 mol^{-1})	χ_t (cm^3 mol^{-1})	τ (s)	α	R
150	0.29200	0.347	0.002530	0.0665	0.000058
300	0.20200	0.347	0.002580	0.0250	0.000059
450	0.13400	0.348	0.002640	0.0253	0.000110
600	0.08840	0.349	0.002680	0.0313	0.000171
750	0.06000	0.348	0.002730	0.0295	0.000123
1000	0.03570	0.348	0.002820	0.0269	0.000117
1250	0.02230	0.347	0.002880	0.0223	0.000118
1500	0.01550	0.346	0.002930	0.0158	0.000095
2000	0.00949	0.346	0.002930	0.0168	0.000141
3000	0.00614	0.342	0.002760	0.0139	0.000099
4000	0.00439	0.337	0.002330	0.0154	0.000040
5000	0.00325	0.331	0.001800	0.0242	0.000090
6000	0.00163	0.324	0.001290	0.0454	0.000110
7000	0.00000	0.317	0.000874	0.0620	0.000284
8000	0.00000	0.307	0.000586	0.0760	0.000456
9000	0.00000	0.297	0.000422	0.0981	0.000044
10000	0.00000	0.285	0.000296	0.0849	0.000385

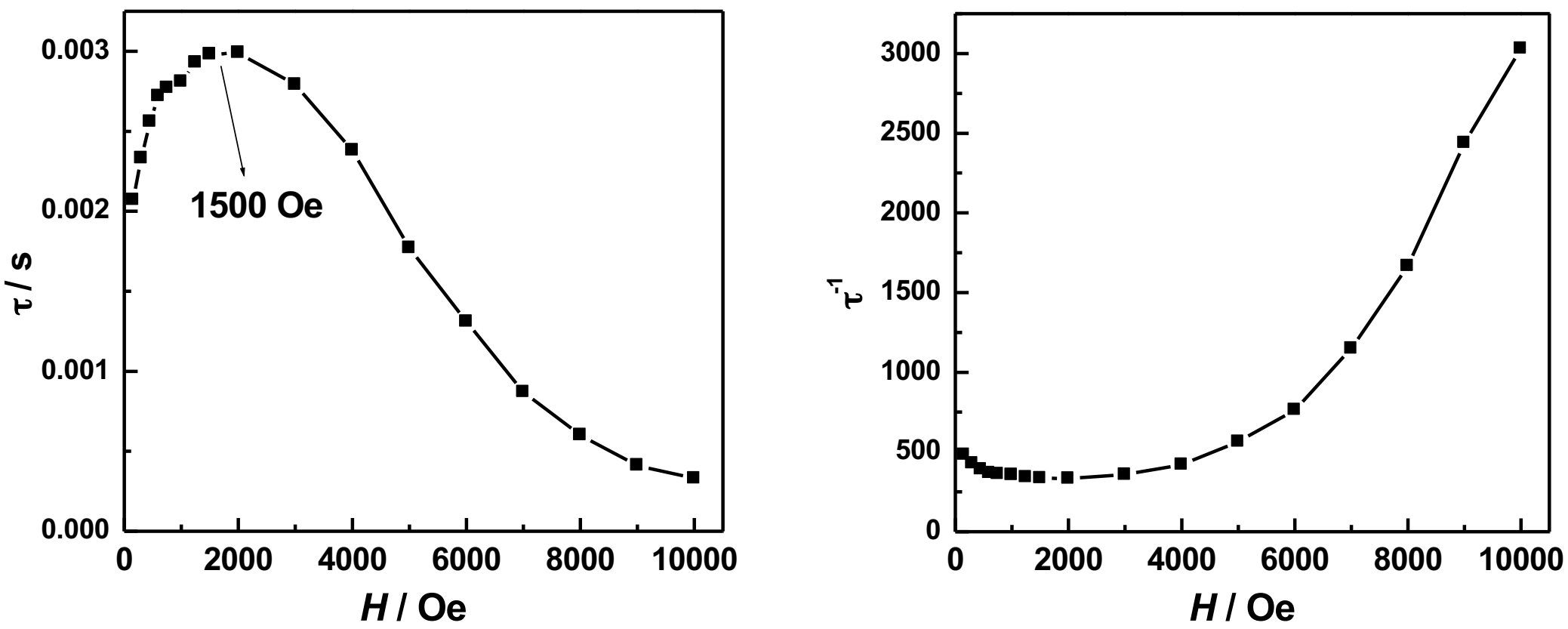

Figure S17. Dependence of τ (left) and τ^{-1} (right) with the applied DC fields for complex **2** at 6.0 K. Solid lines are guides for the eye.

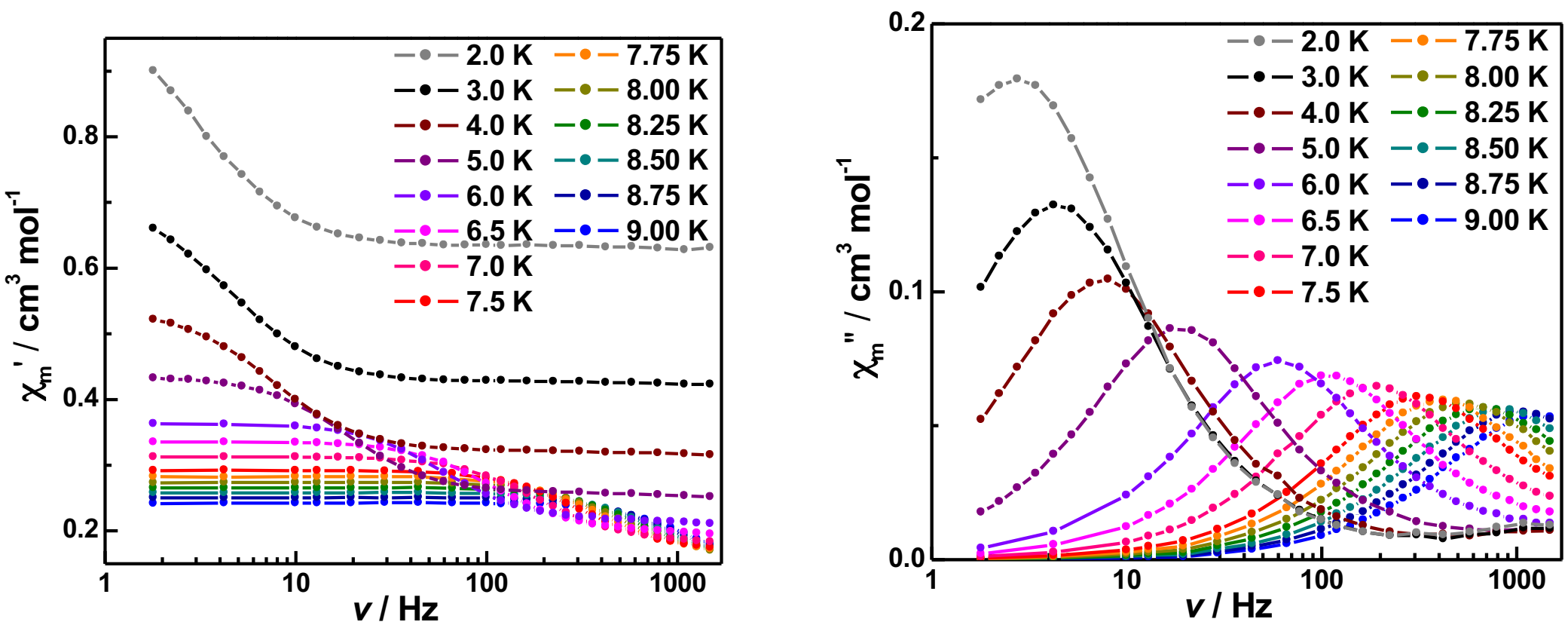

Figure S18. Variable-frequency in-phase (χ_m', left) and out-of-phase (χ_m'', right) components of the ac magnetic susceptibility data for **2**, collected at temperatures from 2.0 to 9.0 K with an ac filed of 5 Oe and 300 Oe dc applied field. Solid lines are guides for the eye.

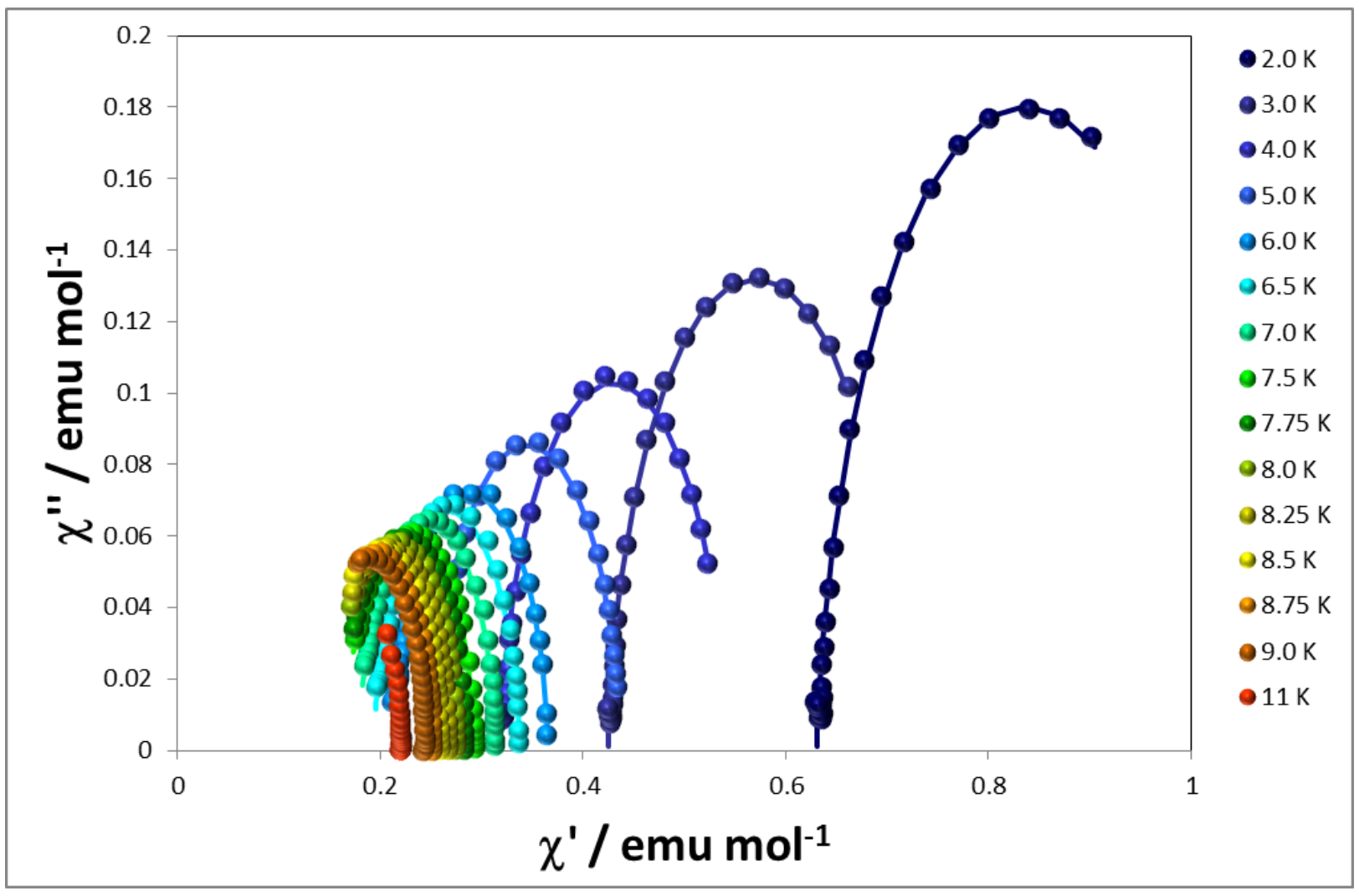

Figure S19. Cole-Cole diagrams of **2** from 2.0 to 11.0 K with an applied dc field of 300 Oe and ac field of 5 Oe. The solid lines are least-square fittings of the data to a distribution of single relaxation processes with a generalized Debye model.

Table S6. Cole-Cole fit values of **2** from 2.0 to 11.0 K, ac field of 5 Oe and dc field of 300 Oe. Data for the temperatures in red are not used in analysis of the dynamic magnetic measurements due to having less than half of the semicircle in the Cole-Cole plot.

T / K	χ_s (cm^3 mol^{-1})	χ_t (cm^3 mol^{-1})	τ (s)	α	R
2.0	0.630	1.040	0.0578000	0.0863	0.0006330
3.0	0.425	0.716	0.0373000	0.0627	0.0004030
4.0	0.319	0.541	0.0216000	0.0492	0.0003550
5.0	0.255	0.436	0.0084500	0.0335	0.0002700
6.0	0.212	0.365	0.0025800	0.0406	0.0002080
6.5	0.194	0.337	0.0014100	0.0381	0.0001550
7.0	0.179	0.314	0.0008150	0.0350	0.0001070
7.5	0.166	0.293	0.0004800	0.0326	0.0000486
7.75	0.161	0.283	0.0003790	0.0222	0.0000259
8.0	0.153	0.275	0.0002940	0.0279	0.0000234
8.25	0.149	0.266	0.0002370	0.0239	0.0000167
8.5	0.143	0.259	0.0001900	0.0204	0.0000151
8.75	0.137	0.251	0.0001530	0.0203	0.0000151
9	0.133	0.243	0.0001270	0.0115	0.0000165
11	0.124	0.220	0.0000422	0.0225	0.0000037

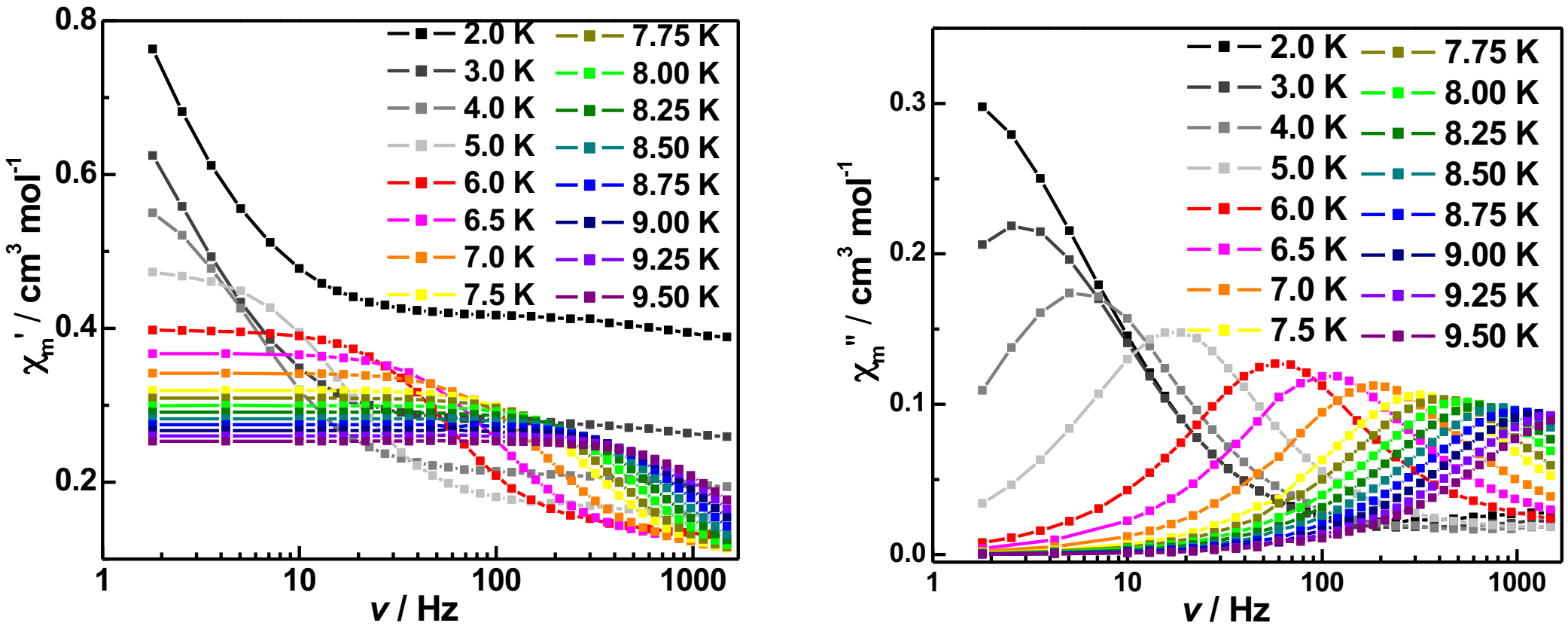

Figure S20. Variable-frequency in-phase (χ_m', top) and out-of-phase (χ_m'', bottom) components of the ac magnetic susceptibility data for **2**, collected at temperatures from 2.0 to 9.5 K with an ac field of 5 Oe and 500 Oe dc applied field. Solid lines are guides for the eye.

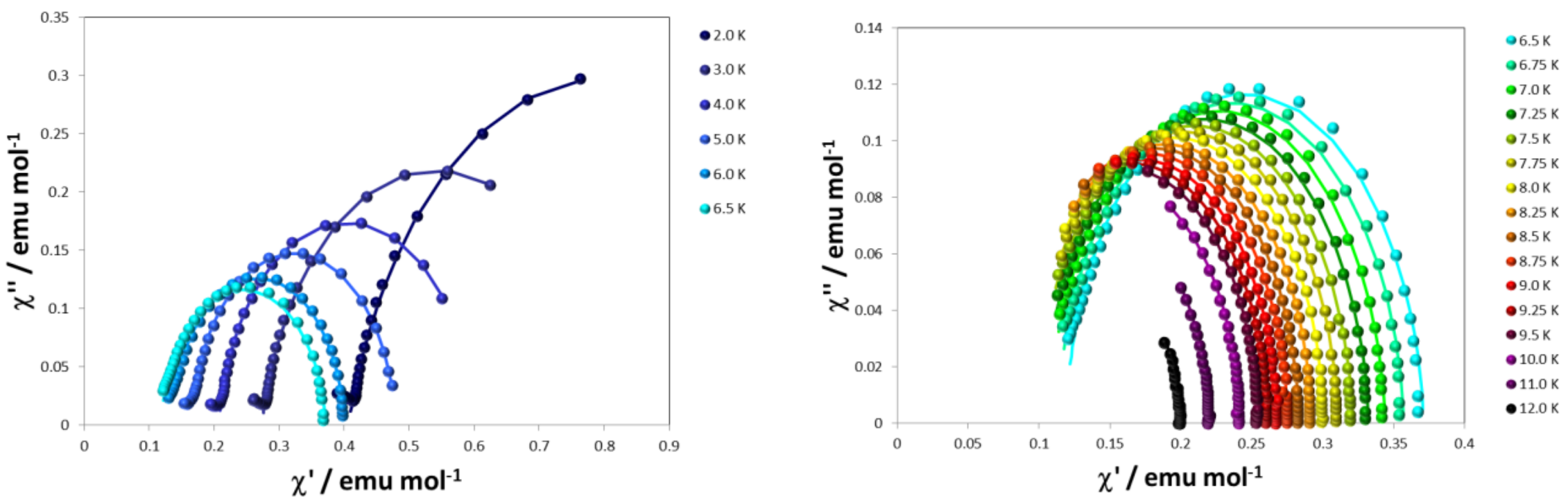

Figure S21. Cole-Cole diagrams of **2** from 2.0 to 12.0 K with an applied dc field of 500 Oe and ac field of 5 Oe. The solid lines are least-square fittings of the data to a distribution of single relaxation processes with a generalized Debye model.

Table S7. Cole-Cole fit values of **2** from 2.0 to 12.0 K, ac field of 5 Oe and dc field of 500 Oe. Data for the temperatures in red are not used in analysis of the dynamic magnetic measurements due to having less than half of the semicircle in the Cole-Cole plot.

T / K	χ_s (cm^3 mol^{-1})	χ_t (cm^3 mol^{-1})	τ (s)	α	R
2.0	0.4070	1.190	0.1010000	0.1750	0.0003590
3.0	0.2730	0.810	0.0570000	0.1270	0.0001510
4.0	0.2070	0.602	0.0276000	0.0772	0.0001290
5.0	0.1670	0.479	0.0092300	0.0352	0.0000892
6.0	0.1350	0.399	0.0026300	0.0286	0.0001950
6.5	0.1180	0.371	0.0014300	0.0507	0.0004960
6.75	0.1130	0.357	0.0010700	0.0475	0.0003430
7.0	0.1070	0.344	0.0008130	0.0427	0.0002310
7.25	0.1030	0.332	0.0006250	0.0392	0.0001590
7.5	0.0980	0.321	0.0004850	0.0343	0.0001240
7.75	0.0941	0.311	0.0003820	0.0302	0.0000659
8.0	0.0892	0.301	0.0003000	0.0264	0.0000471
8.25	0.0857	0.292	0.0002410	0.0256	0.0000251
8.5	0.0809	0.283	0.0001930	0.0227	0.0000186
8.75	0.0771	0.275	0.0001570	0.0217	0.0000190
9.0	0.0754	0.268	0.0001300	0.0168	0.0000180
9.25	0.0704	0.261	0.0001070	0.0208	0.0000096
9.5	0.0680	0.254	0.0000897	0.0194	0.0000131
10	0.0620	0.241	0.0000634	0.0222	0.0000103
11	0.0631	0.220	0.0000373	0.0322	0.0000081
12	0.0881	0.199	0.0000287	0.0403	0.0000119

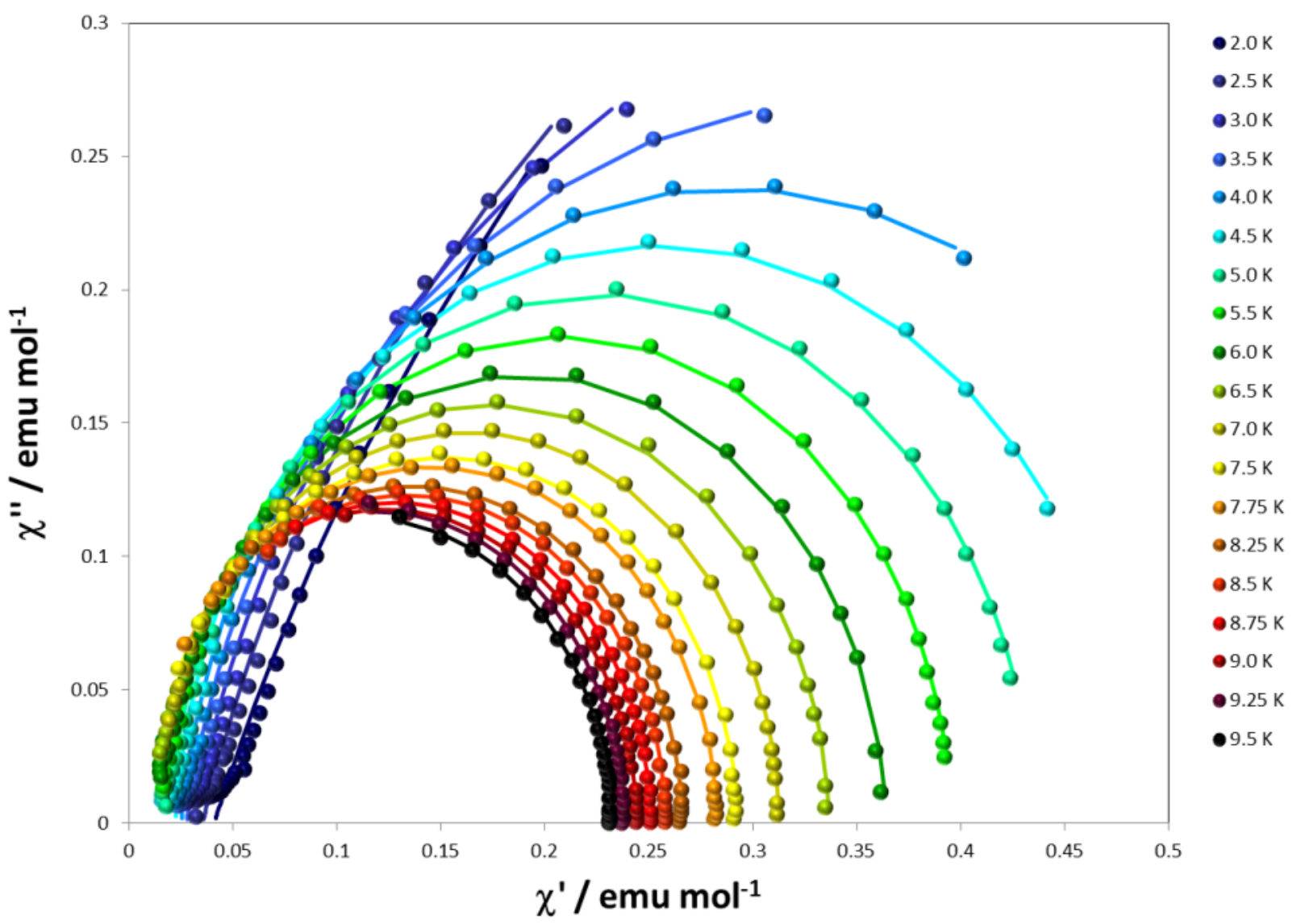

Figure S22. Cole-Cole diagrams of **2** from 2.0 to 9.5 K with an applied dc field of 1500 Oe and ac field of 5 Oe. The solid lines are least-square fittings of the data to a distribution of single relaxation processes with a generalized Debye model.

Table S8. Cole-Cole fit values of **2** from 2.0 to 9.5 K with an ac field of 5 Oe and dc field of 1500 Oe. Data for the temperatures in red are not used in analysis of the dynamic magnetic measurements due to having less than half of the semicircle in the Cole-Cole plot.

T / K	χ_s (cm^3 mol^{-1})	χ_t (cm^3 mol^{-1})	τ (s)	α	R
2.0	0.04100	1.680	0.8050000	0.24900	0.0007510
2.5	0.03550	1.130	0.3590000	0.18900	0.0006700
3.0	0.03090	0.838	0.1960000	0.14600	0.0006510
3.5	0.02760	0.672	0.1090000	0.11000	0.0005990
4.0	0.02530	0.556	0.0531000	0.06890	0.0005810
4.5	0.02260	0.486	0.0249000	0.04320	0.0004840
5.0	0.02040	0.436	0.0117000	0.02950	0.0002970
5.5	0.01860	0.396	0.0057100	0.02040	0.0001930
6.0	0.01540	0.364	0.0029200	0.02330	0.0001290
6.5	0.01380	0.337	0.0015700	0.01820	0.0000905
7.0	0.01290	0.313	0.0008870	0.01480	0.0000607
7.5	0.01020	0.293	0.0005210	0.01830	0.0000709
7.75	0.00894	0.283	0.0004060	0.01580	0.0000816
8.0	0.00696	0.266	0.0002530	0.01450	0.0000209
8.25	0.00917	0.258	0.0002080	0.00637	0.0000606
8.5	0.00504	0.251	0.0001650	0.01440	0.0000090
8.75	0.00221	0.244	0.0001330	0.01820	0.0000469

9.0	0.00000	0.237	0.0001100	0.00890	0.0000254
9.5	0.00000	0.231	0.0000918	0.01030	0.0000241

Analysis of dynamic magnetic measurements.

We attempted to fit the dependence of τ^{-1} with the field using Equation S1, which includes a direct process for Kramers ions and tunneling processes as well as a constant term to include relaxation processes without field dependence. All attempts to fit the dependence of τ^{-1} with the field were unsatisfactory which indicates the complexity of the dependence of the relaxation time with field for compound **1** and **2**.

$$\tau^{-1} = AH^4 + \frac{B_1}{(1 + B_2H^2)} + C \qquad \text{S1}$$

The dependence of τ^{-1} with the temperature at different fields has been analyzed. To facilitate the analysis we have not considered:

- For compound **1**, the values at 3 KOe between 2 and 3.5 K because of the presence of two different relaxation processes.
- For compound **2**, the values at temperatures larger than 9.5 K because the Cole-Cole plots are not a semicircle (less than half of the semicircle) making the fit unrealistic.

The Arrhenius fit gives an energy barrier and pre-expotential factors (τ_0) of 30.6 and 33.6 cm^{-1} / 2.0(2)-3.3(7) · 10^{-7} s at 500 and 3000 Oe for **1** and 42.5(6)-44.7(6) cm^{-1} / 1.0(1)-1.5(2)·10^{-7} s at 500 and 1500 Oe for **2** (Figure S23). From the experimental static magnetic data and from the calculations the obtained D values are -92 and -114 cm^{-1} respectively, which leads to energy differences between states of 184 and 228 cm^{-1} respectively. It is worth noting that an Orbach process should involve a real state and there is not a state around 30 cm^{-1}, which indicates that other relaxation processes are predominant at these temperatures.

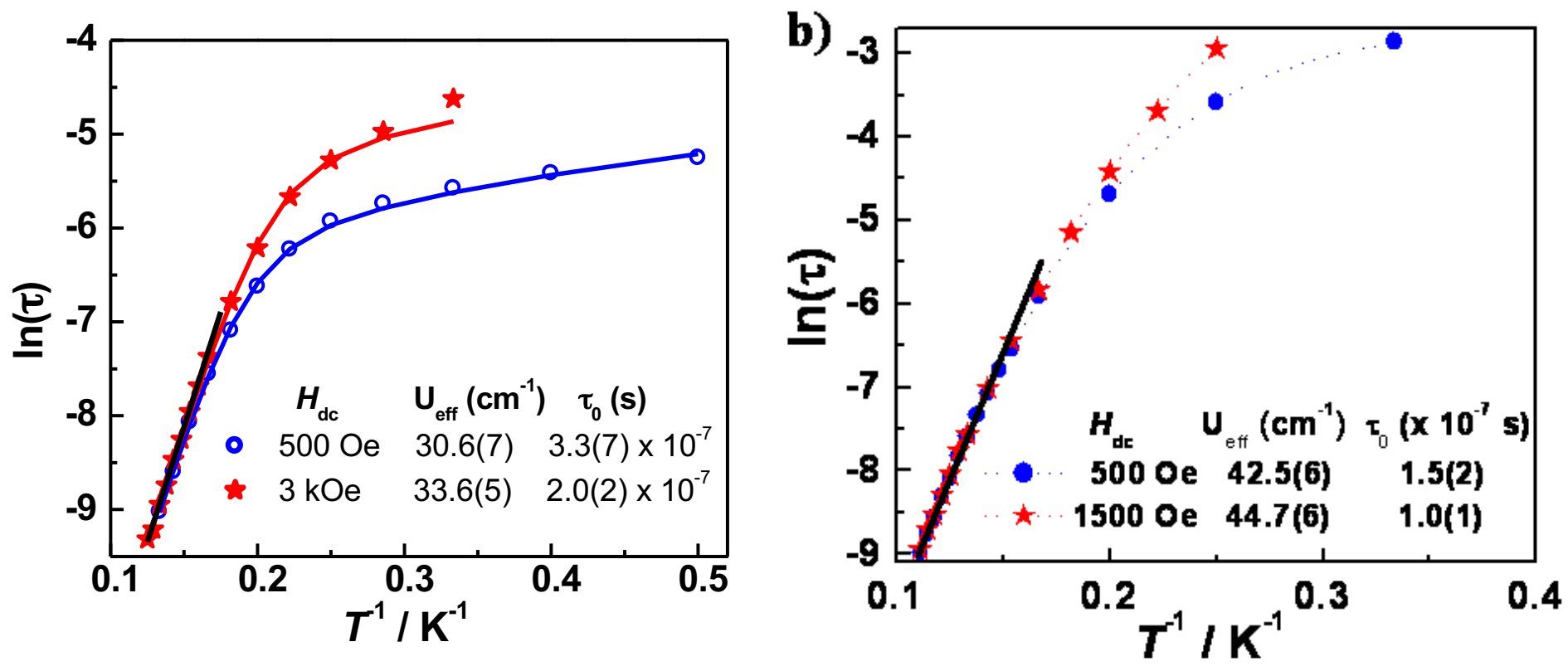

Figure S23. Arrhenius plots of ln(τ) vs T^{-1} for **1** (left) and **2** (right) under different dc fields.

The more common relaxation processes for mononuclear compounds are direct and tunneling (usually predominant at low temperatures), and Raman and Orbach (usually predominant at higher temperatures). A fit including all the possible relaxation pathways (Equation S2) has been performed but the number of parameters are too

high resulting in an over-parametrization of the curves (Figure S24), especially without fixing the direct and tunneling parameters because of the unsuccessful fit of the dependence with the field.

$$\tau^{-1} = ATH^4 + \frac{B_1}{(1 + B_2H^2)} + CT^n + \tau_0{}^{-1}\exp\left(-\frac{U_{eff}}{k_BT}\right) \qquad \text{S2}$$

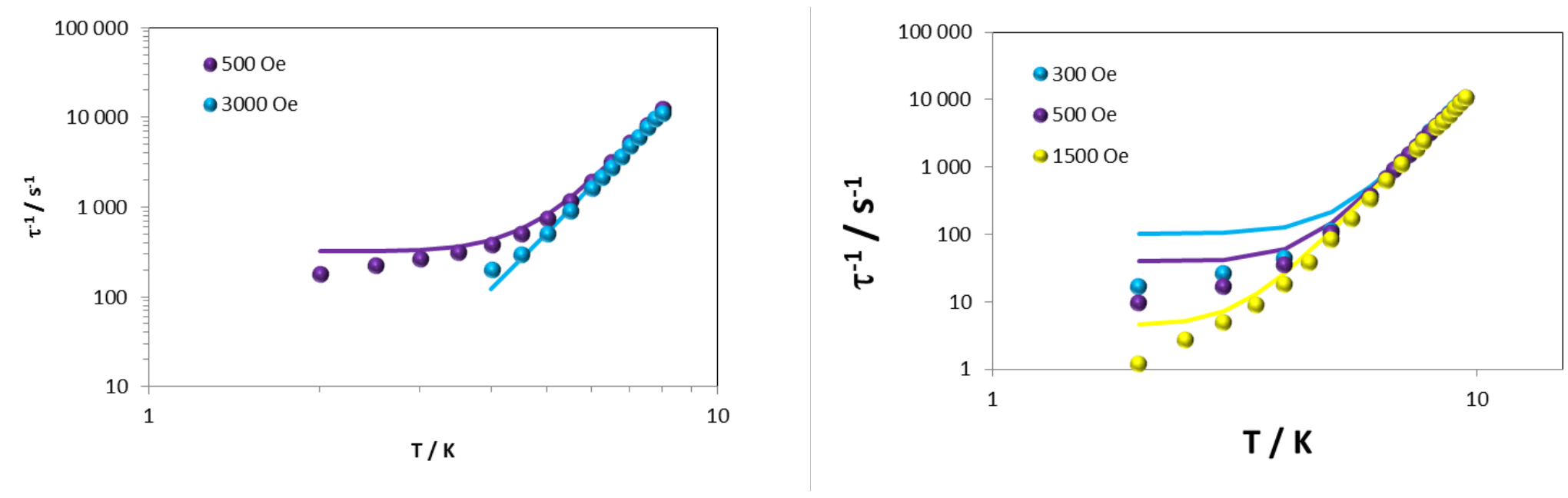

Figure S24. τ^{-1} vs temperature for **1** (left) and **2** (right) at different applied dc fields. The solid line is the best fit obtained using equation S2.

Table S9. Parameters obtained for the fit of the dependence of τ with temperature at different applied dc fields using equation S2 for **1** and **2**.

	**1**	**2**
A / s^{-1} K T^{-4}	0	0
B_1 / s^{-1}	6074.5	1176.5
B_2 / T^{-2}	7247.0	11546.0
C / s^{-1} K^n	0.0098	0.0010
n	6.73	7.20
Ueff / cm^{-1}	205	200
τ_0^{-1}	10000000	10000000

At higher temperatures, Raman or Orbach should be the predominant processes. The large D value gives rise to a large energy difference between states, which should lead to a very slow relaxation time (slower than the measurable relaxation times with the ac measurements possible with our SQUID) allowing us to discard this process at higher temperatures. In fact, when we try to fit the high temperature regime with both processes and an energy barrier of 200 cm^{-1} as a starting parameter, this value remains invariable and the data can be fit exclusively with the Raman term (Figure 25).

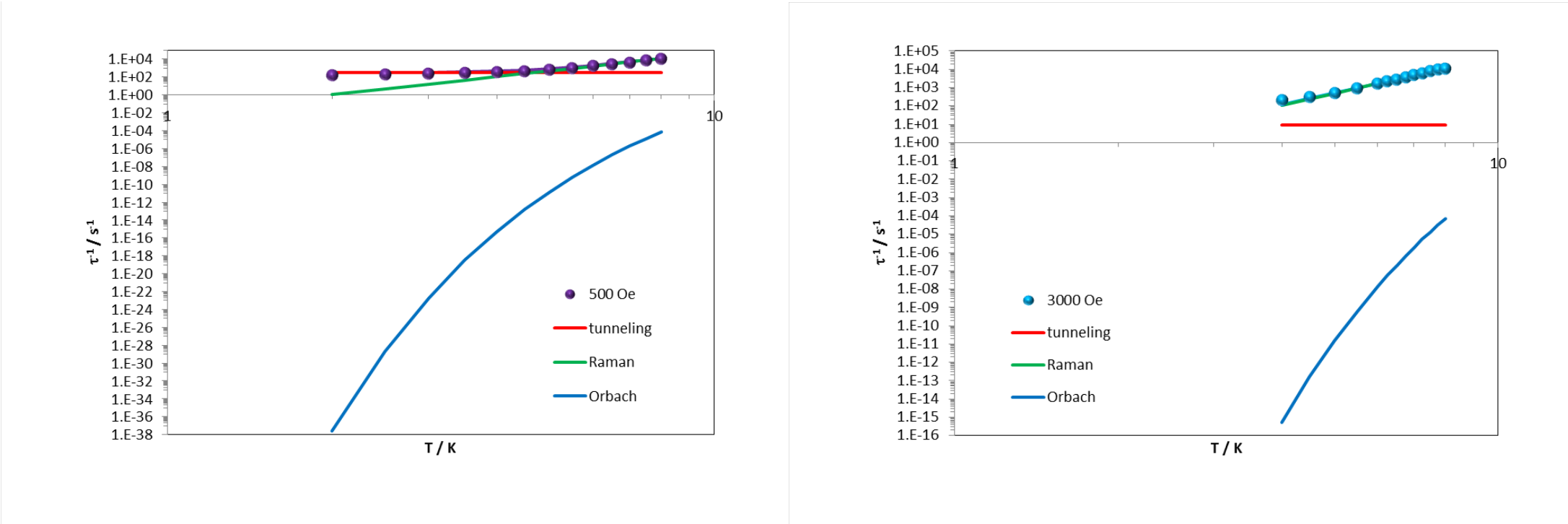

Figure S25. τ^{-1} vs temperature for **1** at 500 Oe (left) and 3000 Oe (right) with the contribution of Orbach, Raman and tunneling processes.

At lower temperatures, direct and tunneling should be the predominant processes. At the applied DC fields (500 and 3000 Oe) τ^{-1} decreases when we increase the field. This indicates that the predominant process should be tunneling because the opposite trend is expected for a direct process (τ^{-1} is proportional to H^4).
After this analysis and to avoid the overparametrization of the curves we decided to fit the dependence of τ^{-1} with temperature using equation 2 and S3.

$$\tau^{-1} = \frac{B_1}{(1 + B_2H^2)} + CT^n \qquad \text{S3}$$

The best fit using equation S3 is shown in Figure S26 with the parameters in Table S10. As can be seen, the low temperature regime is not well described because the error is very small compared with the error produced by the Raman part of the equation.

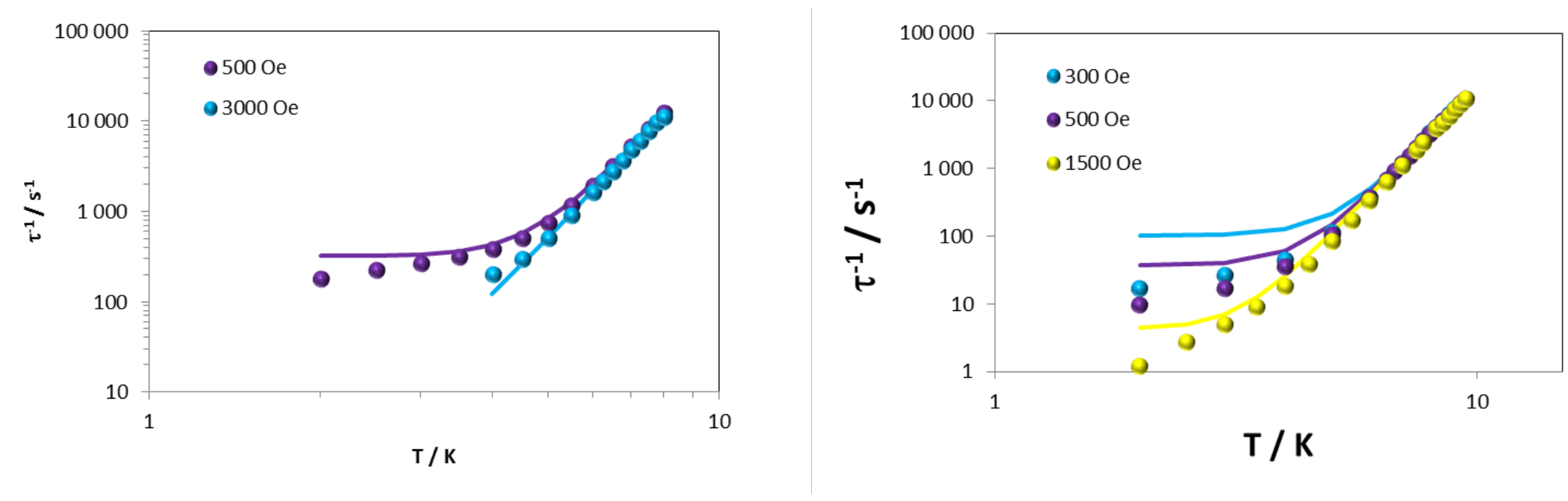

Figure S26. τ^{-1} vs temperature for **1** (left) and **2** (right) at different applied dc fields. The solid line is the best fit obtained using equation S3.

Table S10. Parameters obtained for the fit of the dependence of τ with temperature at different applied dc fields using equation S3 for **1** and **2**.

	**1**	**2**
B_1 / s^{-1}	6963.2	3938.9
B_2 / T^{-2}	8309.2	41251
C / s^{-1} K^n	0.0098	0.0010
n	6.73	7.20

Computational Details.

Table S11. Relative energy (E) in cm^{-1} and Boltzman population at 300 K (BP) of the twelve lowest states computed at CASSCF and NEVPT2 level.

	**1**				**2**			
	**CASSCF**		**NEVPT2**		**CASSCF**		**NEVPT2**	
	**E**	**BP**	**E**	**BP**	**E**	**BP**	**E**	**BP**
**1a**	0.0	0.350	0.0	0.348	0.0	0.349	0.0	0.348
**1b**	0.0	0.350	0.0	0.348	0.0	0.349	0.0	0.348
**2a**	236.3	0.113	232.8	0.114	236.3	0.113	232.8	0.114
**2b**	236.3	0.113	232.8	0.114	236.3	0.113	232.8	0.114
**3a**	508.2	0.0306	509.1	0.0303	504.2	0.0311	503.4	0.0311
**3b**	508.2	0.0306	509.1	0.0303	504.2	0.0311	503.4	0.0311
**4a**	821.5	6.81e-03	809.8	7.17e-03	817.2	6.94e-03	804.1	7.35e-03
**4b**	821.5	6.81e-03	809.8	7.17e-03	817.2	6.94e-03	804.1	7.35e-03
**5a**	2603.5	1.32e-06	3290.3	4.89e-08	2592.3	1.39e-06	3278.0	5.17e-08
**5b**	2603.5	1.32e-06	3290.3	4.89e-08	2592.3	1.39e-06	3278.0	5.17e-08
**6a**	2686.6	8.88e-07	3376.7	3.23e-08	2675.2	9.36e-07	3365.4	3.40e-08
**6b**	2686.6	8.88e-07	3376.7	3.23e-08	2675.2	9.36e-07	3365.4	3.40e-08

Table S12. D and E/D values at CASSCF and NEVPT2 level.

	**1**		**2**	
	CASSCF	NEVPT2	CASSCF	NEVPT2
D (cm^{-1})	-114.54	-114.31	-114.40	-114.23
E/D	0.15	0.11	0.15	0.11

Table S13. Energy of the first five excited states (cm^{-1}) and its main contributions to the D and E values in cm^{-1} at CAS(7,5) NEVPT2 level.

State	Multiplicity	**1**			**2**		
		Energy	D	E	Energy	D	E
Φ_1	4	73.9	-133.9	0.013	53.1	-133.976	-0.034
Φ_2	4	2945.3	8.99	-8.99	2926.3	9.067	-9.071
Φ_3	2	7711.7	2.983	-1.470	12513.7	6.196	1.425
Φ_4	2	7799.1	0.909	0.875	12563.5	-1.871	-1.425
Φ_5	4	12309.5	3.10	-2.52	13714.4	0.560	-1.285

References

S1. *SMART and SAINT*, Siemens Analytical X-ray Instruments Inc., Madison, WI, USA, **1996.**

S2. G. M. Sheldrick, *SADABS*, University of Gottingen, Gottingen, Germany, **1996**.

S3. G. M. Sheldrick, *SHELXS-97 and SHELXL-97*, University of Gottingen, Gottingen, Germany, **1997.**

S4. Diamond - Crystal and Molecular Structure Visualization. Crystal Impact - Dr. H. Putz & Dr. K. Brandenburg GbR, Kreuzherrenstr. 102, 53227 Bonn, Germany
